# Localization of RNAi Machinery to Axonal Branch Points and Growth Cones Is Facilitated by Mitochondria and Is Disrupted in ALS

**DOI:** 10.3389/fnmol.2018.00311

**Published:** 2018-09-05

**Authors:** Noga Gershoni-Emek, Topaz Altman, Ariel Ionescu, Christopher J. Costa, Tal Gradus-Pery, Dianna E. Willis, Eran Perlson

**Affiliations:** ^1^Sagol School of Neuroscience and Department of Physiology and Pharmacology, Sackler School of Medicine, Tel Aviv University, Tel Aviv, Israel; ^2^Burke Neurological Institute, White Plains, NY, United States; ^3^Brain & Mind Research Institute, Weill Cornell Medicine, New York, NY, United States

**Keywords:** axonal transport, microRNA, mitochondria, RISC, ALS, Dicer, local synthesis

## Abstract

Local protein synthesis in neuronal axons plays an important role in essential spatiotemporal signaling processes; however, the molecular basis for the post-transcriptional regulation controlling this process in axons is still not fully understood. Here we studied the axonal mechanisms underlying the transport and localization of microRNA (miRNA) and the RNAi machinery along the axon. We first identified miRNAs, Dicer, and Argonaute-2 (Ago2) in motor neuron (MN) axons. We then studied the localization of RNAi machinery and demonstrated that mitochondria associate with miR-124 and RNAi proteins in axons. Importantly, this co-localization occurs primarily at axonal branch points and growth cones. Moreover, using live cell imaging of a functional Cy3-tagged miR-124, we revealed that this miRNA is actively transported with acidic compartments in axons, and associates with stalled mitochondria at growth cones and axonal branch points. Finally, we observed enhanced retrograde transport of miR-124-Cy3, and a reduction in its localization to static mitochondria in MNs expressing the ALS causative gene hSOD1^G93A^. Taken together, our data suggest that mitochondria participate in the axonal localization and transport of RNAi machinery, and further imply that alterations in this mechanism may be associated with neurodegeneration in ALS.

## Introduction

Neurons are highly polarized cells with well-defined structures such as axons and dendrites. In order to survive and maintain its function, the neuron must be able to respond to both intracellular and extracellular cues. Local protein synthesis is crucial for enabling the cellular response to some cues (Cagnetta et al., 2018). The spatiotemporal localization of mRNAs in distinct intracellular compartments within the neuron, together with the proteins that regulate and execute translation, is thus essential for the neuron's survival and function (Holt and Schuman, 2013). The molecular mechanisms regulating this process have yet to be fully elucidated.

microRNAs (miRNAs) are short, negative regulators of gene expression, which are highly conserved in multicellular organisms. Both pre- and mature miRNAs have been identified in the axonal compartment (Sasaki et al., 2014; Kim et al., 2015; Rotem et al., 2017), suggesting that specifically localized miRNAs and components of the RNA-Induced Silencing Complex (RISC; Hengst, 2006; Zhang et al., 2015) can participate in the regulation of local synthesis in the axon. In the developing axonal growth cone, miRNAs modulate axon growth and guidance in response to extrinsic cues by locally regulating protein translation (Holt and Schuman, 2013). The mechanism underlying miRNA transport and localization has remained elusive for many years (Kosik, 2006; Lemcke and David, 2018).

Mitochondria play multiple, essential roles in both the adult and developing axons. Beyond addressing metabolic needs, mitochondria also serve as important signaling hubs, and are required for local synthesis (Spillane et al., 2013; Merrill and Strack, 2014; Sheng, 2014). This is evident due to the localization of stalled mitochondria in hot spots of translation (i.e., axonal branch points and growth cones), suggesting that mitochondria may also play a role in anchoring factors necessary for the local regulation of protein translation at these sites (Spillane et al., 2013; Cioni et al., 2018). Thus, mitochondrial dysfunction may precede defects in local protein synthesis, which may lead to synapse instability and neurodegeneration.

The association between mitochondria and RNAi machinery has been emerging in the past several years, starting with the discovery that Argonaute-2 (Ago2) associates with mitochondria (Maniataki and Mourelatos, 2005a). This was followed by multiple reports of specific miRNA enrichment in isolated mitochondria (Kren et al., 2009; Bandiera et al., 2011; Barrey et al., 2011; Sripada et al., 2012; Vargas et al., 2016), together with reports that Dicer and Ago2 were identified in isolated mitochondria from hippocampal neurons (Wang et al., 2015; Vargas et al., 2016). Importantly, a two-way functional connection exists between miRNAs and mitochondria. RISC assembly is dependent on ATP production (Yoda et al., 2010), and mitochondria have been shown to associate with P-bodies to modulate RNA interference (Huang et al., 2011). Conversely, miR-338 has a documented function in regulating oxidative phosphorylation, as well as the expression of mitochondrial proteins such as cytochrome C (Aschrafi et al., 2008, 2012). Recently, miR-124 has been shown to regulate mitochondrial activity and localization in MNs (Yardeni et al., 2018).

Amyotrophic Lateral Sclerosis (ALS) is a fatal, progressive, neurodegenerative disease that targets both upper and lower motor neurons (MNs). Among the numerous molecular mechanisms that have been identified in ALS pathology, mitochondrial dysfunction has been gaining interest in recent years as a clinical hallmark of both familial and sporadic ALS (Vandoorne et al., 2018). The most widely studied ALS-related gene, SOD1 has been found to be associated with multiple mitochondrial deficits including impaired mitophagy, ATP generation, calcium buffering, mitochondrial axonal transport, and ROS generation [reviewed in Edens et al. (2016)]. Mitochondrial number (Wiedemann et al., 2002), structure (Siklós et al., 1996), function (Wiedemann et al., 2002), and localization (Sasaki et al., 2007) have all been found to be impaired in ALS.

Alterations in RNA localization and local synthesis can be toxic to the neuron, and have also been associated with neurodegenerative diseases such as ALS, Spinal Muscular Atrophy (SMA), and others (Gershoni-Emek et al., 2015; Walsh et al., 2015; Kapur et al., 2017; Rotem et al., 2017). Recent studies also directly connect ALS pathology with altered global (Kanekura et al., 2016; Russo et al., 2017) and local protein synthesis (Cestra et al., 2017). Similarly, microRNAs have also been implicated in motor neuron degenerative disorders (Williams et al., 2009; Haramati et al., 2010; Rotem et al., 2017). The formation of stress granules in SOD1 and other genetic models (Gal et al., 2016; Maziuk et al., 2017) has been shown to affect protein synthesis in ALS, but a connection between mitochondrial abnormalities and protein synthesis alterations in ALS has not been established.

Here, we first demonstrate the localization of miRNAs, Dicer, and Ago2 in distal axons and growth cones in cultured primary MNs, as well as in sciatic nerve sections and the neuromuscular junction (NMJ). We then use immunofluorescence to detect colocalization of mitochondria with the neuronal miR-124 and Dicer in translation-enriched areas such as axonal branch points and growth cones. Next, we use a fluorescently tagged miR-124-Cy3 mimic to study the cellular transport and localization of this miRNA in mutant SOD (mSOD) and wild type (WT) neurons, and its association with mitochondria. Our data indicate that miR-124-Cy3 is actively transported with acidic compartments, and that in translation-enriched areas it colocalizes with static mitochondria. In MNs cultured from ALS mutated mSOD1 mice, we detect a shift in the localization of miR-124-Cy3 from static to mobile mitochondria whereas the retrograde axonal transport of miR-124 is increased. Our data suggest a model in which mitochondria takes part in the regulation of local protein synthesis in growth cones and axonal junctions by anchoring RISC proteins and miRNA, a process that is disrupted in mSOD1 neurons, thus possibly contributing to the alterations in local synthesis observed in SOD1 mutants.

## Materials and methods

### Mice

hSOD1^G93A^ mice (Gurney et al., 1994) were originally obtained from Jackson Laboratories, and the colony was maintained by breeding with C57BL/6J mice.

HB9::GFP mice were originally obtained from Jackson Laboratories, and the colony was maintained by breeding with ICR mice. Pregnant ICR mice were obtained from the Institute of Animal Science, Harlan. All animal experiments were approved by the Animal Ethics Committee of Tel-Aviv University. Primary cultures were harvested from mouse embryos of either sex.

### Cell culture

#### Cell lines

The human embryonic kidney 293T (HEK293T) cell line was maintained in culture medium containing DMEM (Biological Industries, Beit HaEmek, Israel), 1% vol/vol Glutamax (Gibco), 1% vol/vol Penicillin/Streptomycin, and 10% Fetal Bovine Serum (Biological Industries). Cells were grown at 37°C in 5% CO_2_.

#### Primary motor neuron culture

Primary MNs were cultured from E12.5 mouse embryos as previously described (Camu and Henderson, 1994; Kalmar and Greensmith, 2009). Briefly, spinal cords were excised, trypsinized, and triturated. Supernatant was collected and centrifuged through a BSA cushion. The pellet was resuspended and centrifuged through an Optiprep gradient (10.4% Optiprep (Sigma), 10 mM Tricine, 4% Glucose) for 20 min at 760 × g with the brake turned off. Next, the cells were collected from the interphase, washed once in complete medium, and then plated in coated growth chambers. Cells were maintained in Complete Neurobasal Medium (Gibco) containing B27, 10% (vol/vol) horse serum, 25 μM beta-mercaptoethanol, 1% Penicillin-Streptomycin (Biological Industries), and 1% Glutamax (Gibco) supplemented with 1 ng/ml GDNF, 0.5 ng/ml CNTF, and 1 ng/ml BDNF (all from Alomone Labs). Prior to seeding, growth chambers, including cover slides and inserts (FAL353102, Falcon), were coated with 1.5 μg/ml poly DL-ornithine (Sigma) overnight at 37°C, and 3 μg/ml Laminin (Sigma) for 2h at 37°C. For immunofluorescence staining, 5 × 10^4^ or 10^4^ cells were seeded on cover slides in 24-well plates. On a “Modified Boyden chamber,” 5 × 10^5^ cells were seeded and grown for 10 days. Cells were grown at 37°C in 5% CO_2_.

For mutant SOD1 (mSOD1) MN culture, embryos were obtained from pregnant WT C57BL/6J females crossed with heterozygous mSOD1 males. Excised spinal cords were maintained in a solution of L15 medium containing 5% Fetal Calf Serum and 1% Penicillin-Streptomycin at 37°C while PCR genotyping was performed. Spinal cords were then pooled and cultured as described. The genotyping primers used were hSOD1^G93A^-forward primer CAT CAG CCC TAA TCC ATC TGA, and hSOD1 ^G93A^-reverse primer CGC GAC TAA CAA TCA AAG TGA.

#### Microfluidic chamber preparation

Polydimethylsiloxane (PDMS) microfluidic chambers (MFC) were designed as described in Gluska et al. (2014) and Zahavi et al. (2015). Briefly, MFCs for dissociated cell culture were punctured with 6 mm wells at each end of the channels surrounding the grooves. Two 6 mm wells were punctured into both ends of the “distal” or axon channel to allow one to control the distal channel. Microfluidic devices were cleaned of surface particles using adhesive tape and sterilized in 70% high-grade ethanol for 1 h. These devices were allowed to completely air dry under sterile conditions, and then attached to sterile 35 mm glass bottom dishes (WPI-FD35-100, WPI) using gentle pressure and heated to 60°C for 20 min to improve their adhesion to glass.

#### MN in microfluidic chambers

Chambers were coated using 1.5 μg/mL poly DL-ornithine and 3μg/mL Laminin (both from Sigma) in DDW overnight at room temperature, and then replaced with complete medium 2 h prior to seeding and finally incubated at 37°C. About 10 × 10^4^ cells were seeded in the proximal channel in 4 μL Neurobasal complete (supplemented with 2% B27 and 50 ng/mL BDNF) and allowed to settle. After 1 h, 100μL BDNF-enriched medium was added to each well. Two days later, medium in the proximal compartment was changed to complete neurobasal medium, whereas the distal compartment was supplemented with 25 ng/mL BDNF-enriched medium. Cells were grown for another 2–4 days until axons traversed the distal compartment before staining and imaging.

#### “Modified boyden chamber” membrane platform and axonal harvesting

Membrane inserts with 1 μm pores (Falcon® Permeable Support for 6-well plates features a transparent PET membrane with 1.0 μm pores, Corning) were placed in a 6-well plate (Corning). The membranes were coated first by filling the membrane insert (2 mL) and the corresponding well (3 mL) with poly-DL-ornithine (1.5 μg/mL, Sigma-Aldrich) overnight at 37°C then with Laminin (3 μg/mL, Sigma-Aldrich) for 2 h at 37°C prior to cell plating. Neurons were then plated on the coated membrane inserts (500,000 cells per insert). After 10 DIV, each culture was washed by gently replacing the growth medium with PBS warmed to 37°C. The remaining wells were subjected to two further 37°C PBS washes. After the third wash, PBS was aspirated away, and the bottom part of the insert (axonal domain) was scraped using a sterile swab tip (TX761MD, Tex-wipe). The scraping tip of the swab was then cut and placed into TRI Reagent (Sigma), for 1–2 min, before the TRI reagent was transferred into a new collection tube for RNA extraction.

### RNA extraction and RT-PCR

RNA was extracted using TRI Reagent (Sigma), and then reverse transcribed using the SuperScript II reverse transcription kit (Life Technologies). RT-PCR was performed with KAPA ReadyMix using the following primers (synthesized either by HyLabs or by Syntezza): PolB forward primer, CCAAGGACAGGAGTGAATGAC, PolB reverse primer, AAGCACAGAGAAGAGGCAATC, Beta-actin forward primer, GTATGGAATCCTGTGGCATC, Beta-actin reverse primer, and AAGCACTTGCGGTGCACGAT. For miRNA detection, cDNA was reverse transcribed using the QuantiMir RT Kit (SBI), and RT-PCR was performed with the SBI universal reverse primer and the following primers: mir-124, TAAGGCACGCGGTGAATGCC, miR-9, TCTTTGGTTATCTAGCTGTATGA, miR-206, TGGAATGTAAGGAAGTGTGTGG, miR-155, and TTAATGCTAATTGTGATAGGGGT. PCR products were run on 2% agarose gel.

### *In situ* hybridization

*In situ* hybridization was performed with Exiqon fluorescein isothiocynate conjugated miRNA LNA probes following the protocol of Lu and Tsourkas (2009; 2011) A scrambled probe was used as a negative control. Briefly, MN were grown on 13 mm coverslips and fixed with 4% vol/vol PFA in PBS for 30 min at room temperature, then washed three times with PBS. Cells were then permeabilized with 70% ethanol overnight at 4°C, and then washed again with PBS. Coverslips were pre-hybridized by incubating with ISH buffer for 2 h in a humid chamber at 60°C. Hybridization buffer was prepared by adding LNA probes to the buffer, heated at 85°C for 5 min, then chilled on ice for 5 min. Pre-hybridization buffer was replaced by hybridization buffer and slides were incubated in a humid chamber at 55°C for 3 h. Slides were washed briefly with 4X SSC at 37°C, followed by 30 min in 2X SSC, 30 min in 1X SSC, and 20 min in 0.1X SSC, all at 37°C. The LNA signal was then amplified using the TSA Plus Fluorescein kit (PerkinElmer) following the manufacturer's instructions. Coverslips were then blocked in PBS + 5% normal goat serum and 5% normal donkey serum for 1 h at room temperature.

### Immunofluorescent staining for cells

Primary MN were grown on 13 mm glass cover slides, and then fixed in 4% paraformaldehyde (PFA) for 10 min at room temperature. Cover slides were then washed in PBS and permeabilized with 0.5% Triton in blocking solution containing 5% donkey serum (Jackson Laboratories) and 1 mg/mL BSA (Sigma) for 5–30 min. After three washes, cells were incubated with appropriate antibodies overnight at 4°C in blocking solution [rabbit anti-Dicer (Abcam,1:100), rabbit anti-Ago2 (Abcam,1:200), chicken anti-NFH (Abcam and Millipore, 1:1000), mouse anti-neurofilament phosphorylated (SMI31)/non-phosphorylated (SMI32) (Covance, 1:500), mouse anti-Rab7 (Abcam, 1:300), mouse anti-TrkB (BD Bioscience, 1:200), mouse anti-COX IV (1:200; Cell Signaling Technology)]. After having been washed, secondary antibodies (2 μg/mL Jackson Laboratories, ThermoFisher) in blocking solution were added for 2 h at room temperature. Phalloidin was used to visualize F-actin, and added when necessary to the secondary antibody. For axonal visualization, cells were incubated for 45 min with Alexa Fluor 594/405-conjugated phalloidin (1x, Abcam), diluted in PBS, then washed and mounted with ProLong Gold (Life Technologies). For mitochondria staining, the cells were incubated with Mitotracker Deep Red FM (100 nM, Thermo Fisher) for 30 min at 37°C, and then washed 3 times with PBS prior to fixation.

### NMJ staining

Gastrocnemius was excised from adult mice and cleared of connective tissue, washed in PBS, fixed in 4% PFA, washed again, and finally incubated with 1 μg/mL Rhodamine Red-conjugated Bungarotoxin (Sigma). Tissues were washed, and then treated with MeOH at −20°C for 5 min, and then washed and blocked in blocking solution for 1 h. Next, the tissues were rocked with appropriate primary antibodies diluted in blocking solution at room temperature overnight. After having been washed, secondary antibodies were added for 4 h at room temperature. Muscle fibers were spread into monolayers under a stereomicroscope and affixed to slides using VectaShield (Vector Laboratories). Cover slides were sealed with clear nail polish.

### Immunofluorescent staining for cryosections

Sciatic nerves and spinal cords were excised from adult mice, washed, and then fixed with 4% vol/vol PFA for 30 min at 4°C. After having been washed, tissues were transferred to PBS with 15% Sucrose for 4 h at 4°C, then to PBS with 30% sucrose overnight at 4°C. Tissues were then affixed in Tissue-Tek OCT compound (Sakura) and frozen in liquid nitrogen, then sectioned using a cryostat and stained as described above.

### Live cell imaging

Live cell imaging was performed under 5% CO_2_ humidified conditions, at 37°C on a laser spinning-disc confocal attached to a Nikon Ti equipped with an Andor iXon 897 EMCCD camera.

### miRNA-124-Cy3 construct and live cell imaging dyes

A fluorescent miR-124-Cy3 construct was designed by Mark Behlke from IDT and purchased from Syntezza. The probe was transfected into MN using Lipofectamine 2000 (Life Technologies) at a concentration of 800 ng DNA per 100,000 cells (=1 MFC), and incubated for 4 h at 37°C, then washed with complete neurobasal medium and stained for mitochondria [100 nM MitoTracker Deep Red FM (Life Technologies)] and acidic compartments [100 nM LysoTracker Green (Life Technologies)] for 30 min followed by 3 washes with complete medium. For imaging, 100 frames were collected at 3-s intervals.

### Protein extraction

Brain, spinal cords, and muscles were excised from adult mice. If not used immediately, they were flash frozen in liquid nitrogen and stored at −80°C until used. Lysis buffer consisting of PBS with 1% Triton X-100 and protease inhibitors (Roche) was then added, and tissues were cut, and then crushed with a manual homogenizer. After 30 min of incubation on ice, samples were centrifuged at 17,000 × g and supernatant was transferred to a clean tube. Protein concentration was determined using a Bio-Rad Protein assay. Cultured cells were collected into lysis buffer with protease inhibitors (Roche). NP40 lysis buffer consisted of PBS with 1% NP40, 50 mM Tris-HCl pH = 7.5 and 150 mM NaCl. RIPA lysis buffer consisted of PBS with 50 mM Tris-HCl pH = 7.5, 150 mM NaCl, 0.5% SDS, 0.5% deoxycholic salt, and 1% NP40. Samples were mixed well and incubated on ice for 30 min, then centrifuged at 17,000 × g for 15 min and the supernatant was transferred to a clean tube. Protein concentration was determined using Bio-Rad Protein Assay (Bio-Rad Laboratories) according to the manufacturer's instructions.

### Collection of sciatic axoplasm

Sciatic nerves were excised from adult mice and placed in cold PBS with protease inhibitors, cut and then gently crushed to extract the axoplasm. The sample was then centrifuged at 17,000 × g and the supernatant was transferred to a clean tube. Protein concentration was determined using Bio-Rad Protein Assay reagent (Bio-Rad Laboratories) according to the manufacturer's instructions.

### Western blotting

Protein samples were denatured by boiling in SDS sample buffer, and then electrophoresed in 7.5%, 10%, or 12% polyacrylamide gels. Proteins were transferred to a nitrocellulose membrane, then immunoblotted with appropriate primary antibodies [Dicer1 (Abcam, 1:500), Ago2 (Abcam, 1:500), Tau (Abcam, 1:500), α-tubulin (Abcam, 1:5000), GFP (Covance, 1:300), gERK (ERK 1/2, Sigma, 1:20000)] followed by HRP-conjugated secondary antibodies (1:10000, Jackson Laboratories) and visualized using myECL Imager (Thermo), according to the manufacturer's instructions. Quantification was performed using Image J software.

### Image analysis

Colocalization measurements were obtained from three-dimensional image stacks, using Imaris Coloc (Bitplane, Inc.). The image was masked for the NFH channel and the colocalization values, reflecting the percentage of colocalized volume per channel, were calculated. The values from several images were averaged and were presented as the percentage of channel A (e.g., Dicer, miR-124-Cy3) localized to channel B (e.g., Rab7, acidic compartments, mitochondria). Growth cone and axonal branch points were determined as a distance of <10 μm from the visible end of the axon, or a diameter of 20 μm surrounding an axonal branch point.

Live cell data were analyzed using the Kymo ToolBox plug-in in ImageJ to generate and analyze kymographs (Zala et al., 2013). A kymograph was generated from each channel separately, and colocalization was determined where the miR-124-Cy3 track overlapped with a track in the corresponding kymograph. For validation of true colocalization compared to random, one of the channels was flipped horizontally and then vertically and the tracks compared.

### Statistical analysis

Statistical analysis was performed using IBM SPSS Statistics v.22. Values are presented with S.E.M. For two-group comparisons, Student's *t*-test was used. For multiple comparisons, ANOVA with Dunnet's or Tukey's *post-hoc* test was applied. Significance was set at *p* < 0.05. All averaged and S.E.M values represent pulled results from three or more independent experiments.

## Results

### miRNAs are localized to MN distal axons and growth cones

**Figure 1:**
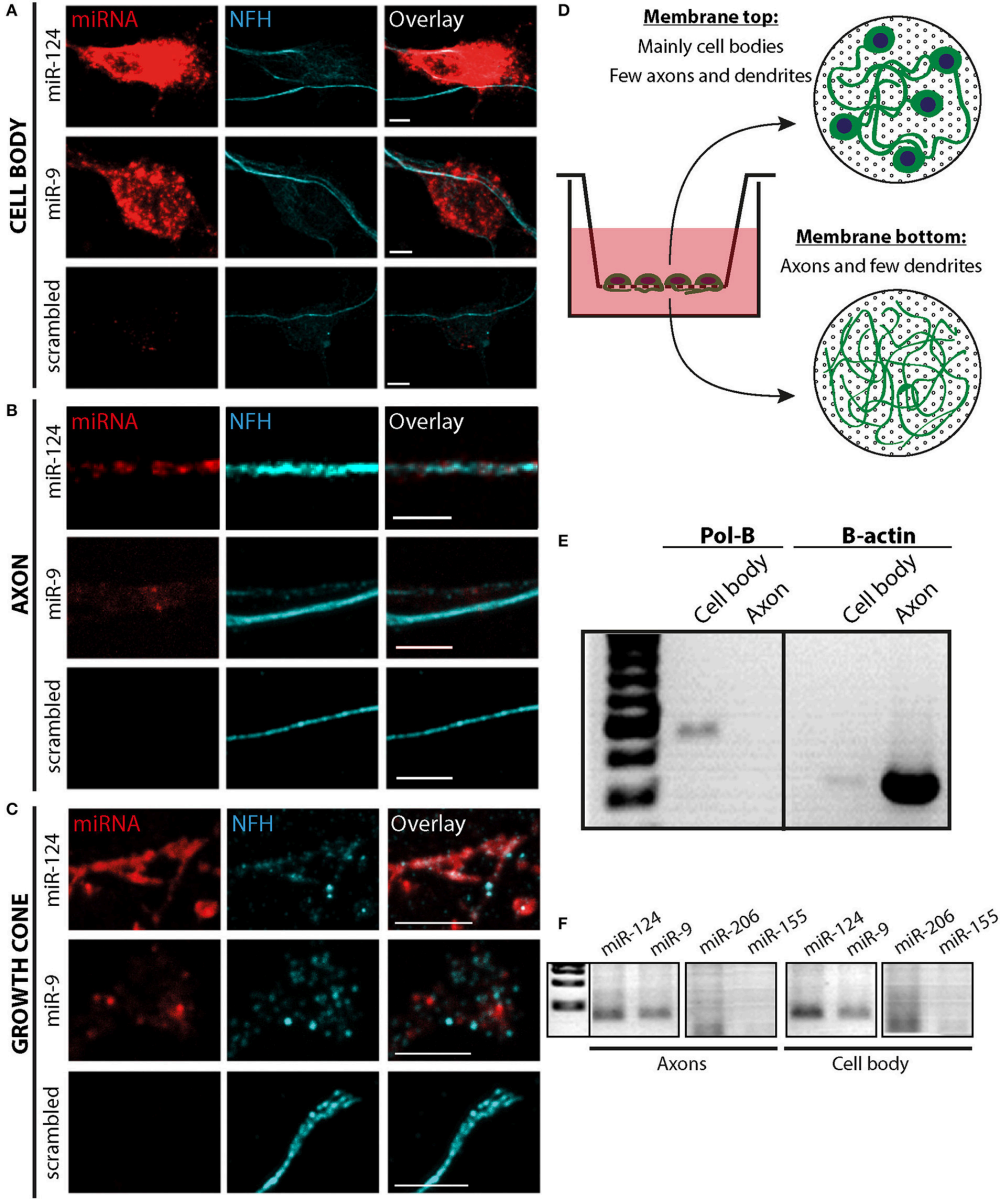
miRNA localization in distal axonal compartments. **(A–C)** *In-situ* hybridization for neuron-specific miR-9 and miR-124 in MN cultures detected these miRNAs in a punctate pattern in the cell body **(A)**, along neurites **(B)**, and in the distal growth cone compartment **(C)**. As a control, a scrambled probe was used. Scale bars = 5 μm. **(D–F)** Membrane-based compartmental platform for culturing of primary MNs. MNs are seeded on inserts. After 10 days axons traverse to the lower side of the membrane, as depicted in the illustration **(D)**, and material from the upper and lower membranes was collected separately for RNA extraction. **(E)** Qualitative RT-PCR was performed for cell body-specific Polymerase B (Pol-B) as a quality control for fraction purity, together with axonal enriched Beta-Actin as a control. **(F)** Neuron-specific miRNAs miR-124 and miR-9 were detected in both neuronal compartments by qualitative RT-PCR, whereas the non-neuronal controls, miR-206 and miR-155, were not.

We first sought to identify miRNAs in primary MNs *in vitro*. We chose to focus on two well-characterized neuronal miRNAs: miR-124 and miR-9 (Gao, 2010). Fluorescent *in situ* hybridization (FISH) was used to detect the localization of these miRNAs in the cell body (Figure 1A), axon (Figure 1B), and growth cone (Figure 1C). In order to supplement these results, we cultured MNs on a porous membrane (“Modified Boyden Chamber”) that enabled us to separate the axons from the cell bodies (Figures 1D,E; Gumy et al., 2011; Rotem et al., 2017). RNA from the upper and lower membrane was harvested, followed by RT-PCR analysis. Interestingly, the neuronal miRNAs miR-124 and miR-9 were readily detected in both axons and cell bodies (Figure 1F), whereas the non-neuronal miRNAs miR-155 and miR-206 were not. Thus, neuronal miRNAs can be localized not only in cell bodies but also in axons and growth cones of primary MN cultures.

### RISC proteins dicer and Ago2 are localized to distal neuronal compartments

**Figure 2:**
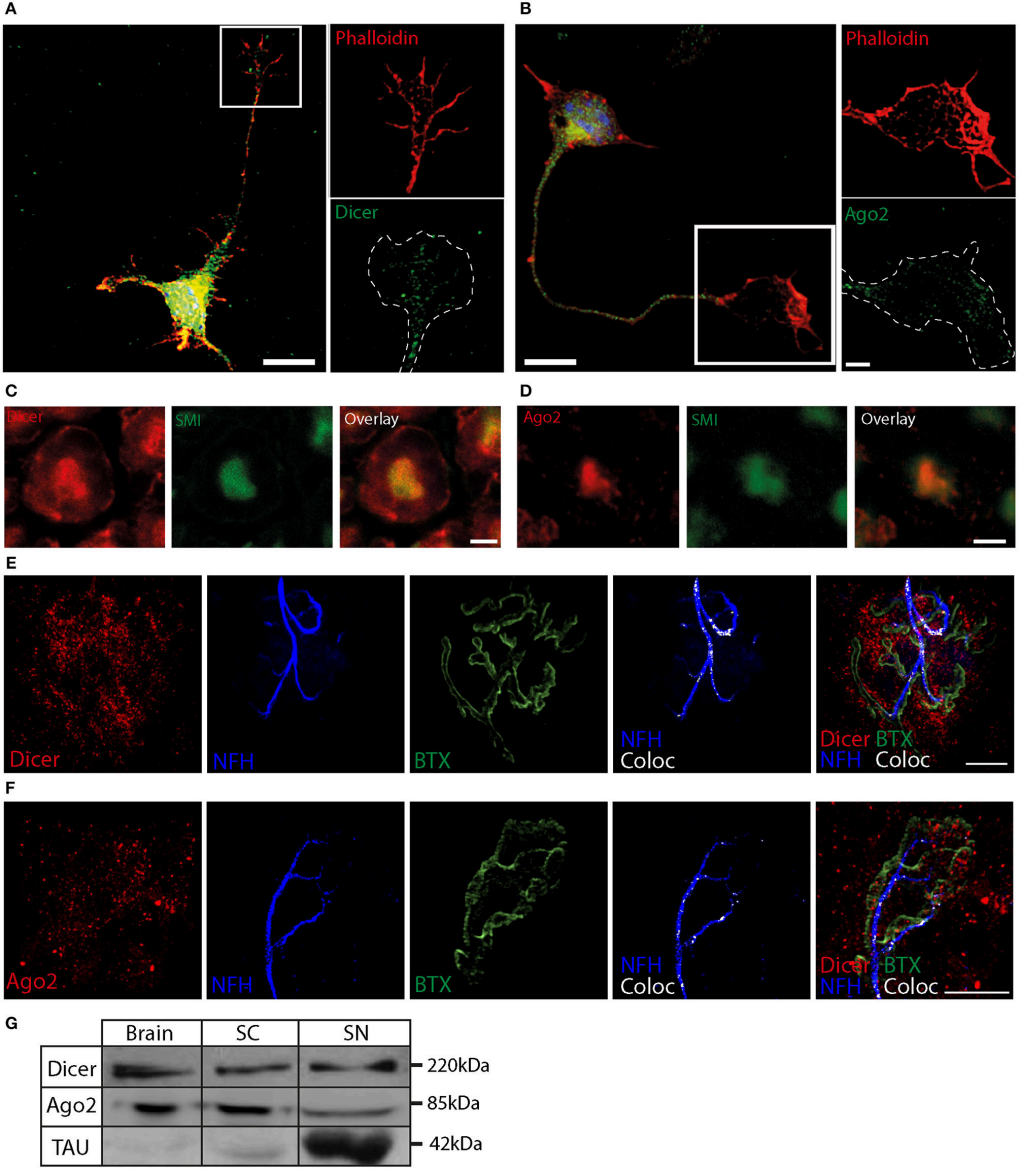
RNAi machinery localization in distal neuronal compartments **(A,B)** Dicer **(A)** and Ago2 **(B)** can be detected in a punctate pattern in the cell body and in the axonal growth cone of fixed MNs. The blue in overlay images indicates a DAPI nuclear stain. The white dashed line represents phalloidin borders. **(C,D)** Mouse sciatic nerve was sectioned into 10 μm slices and stained for Dicer **(C)**, Ago2 **(C)**, and neurofilament (SMI). Scale bar = 3 μm. **(E,F)** NMJ of adult mice shows that both Dicer **(E)** and Ago2 **(F)** are enriched in the vicinity of the NMJ, and are partially colocalized with presynaptic NFH marker (white color indicates 3D-colocalization channel of Dicer or Ago 2 with NFH). Scale bar = 10 μm. **(G)** Western blot identifies full-length Dicer at ~220 kDa, Ago2 at ~85 KDa, and the axonal marker tau at ~42 KDa in mouse brain, spinal cord (SC), and sciatic nerve axoplasm (SN).

We then investigated whether the proteins involved in miRNA function are localized at distal parts of the neuron, indicating a spatial, functional role for these miRNAs. To this end, we focused on Dicer, which plays a role in loading the miRNAs into RISC, in addition to its role in pre-miRNA cleavage (Maniataki and Mourelatos, 2005b; MacRae et al., 2008), and on Ago2, the core component of RISC (Kawamata and Tomari, 2010). Primary MN were grown for three days in culture to enable a short axon to grow with a visible growth cone, cultures were fixed and immunostained for Dicer and Ago2. Dicer and Ago2 display a similar, punctate pattern of expression along all neuronal compartments including the axon and the growth cone (Figures 2A,B; Supplementary Figure 1). This pattern is similar to previously described patterns for Dicer and Ago2 in distal compartments of large neurons (Lugli et al., 2005; Hengst, 2006), suggesting that RISC is localized to the growth cones.

After validating the localization of miRNAs and the RNAi machinery in distal axonal compartments *in vitro*, we next investigated whether this distal localization also occurs in adult mice *in vivo*. For this purpose, mouse sciatic nerves were sectioned into 10 μm transverse slices, and stained for Dicer and Ago2, which can be seen localized to the NFH stain in the axons (Figures 2C,D). Dicer is also abundant in the surrounding Schwann cell.

In adult mice, the most distal part of the motor neuron axon can be found in the NMJ, a specialized synapse structure comprising the motor neuron axon, the muscle, and the terminal Schwann cells. In order to detect Dicer and Ago2 in these structures, we performed whole-mount staining of the gastrocnemius leg muscle to visualize Dicer and Ago2 (Figures 2E,F; Supplementary Figure 2) together with Bungarotoxin (BTX) stained post-synaptic nicotinic Acetylcholine receptor (nAChR) aggregation and pre-synaptic neurofilament accumulation. Our results indicate an enrichment of both proteins around the NMJ, which would be expected in an area of high activity. A closer look at the analyzed co-localization images reveals that some of the observed puncta are pre-synaptic and localized to the neurofilament. Consistent with our immunostaining results, western blot analysis demonstrates that Dicer and Ago2 are found at comparable levels in the sciatic nerve axoplasm, brain, and spinal cord (Figure 2G). Thus, the RISC components, Dicer and Ago, are found *in vivo* in distal axons and synapses of adult mice.

### Localization of miRNA and RNAi machinery to mitochondria is enhanced in growth cones and at axonal branch points

**Figure 3:**
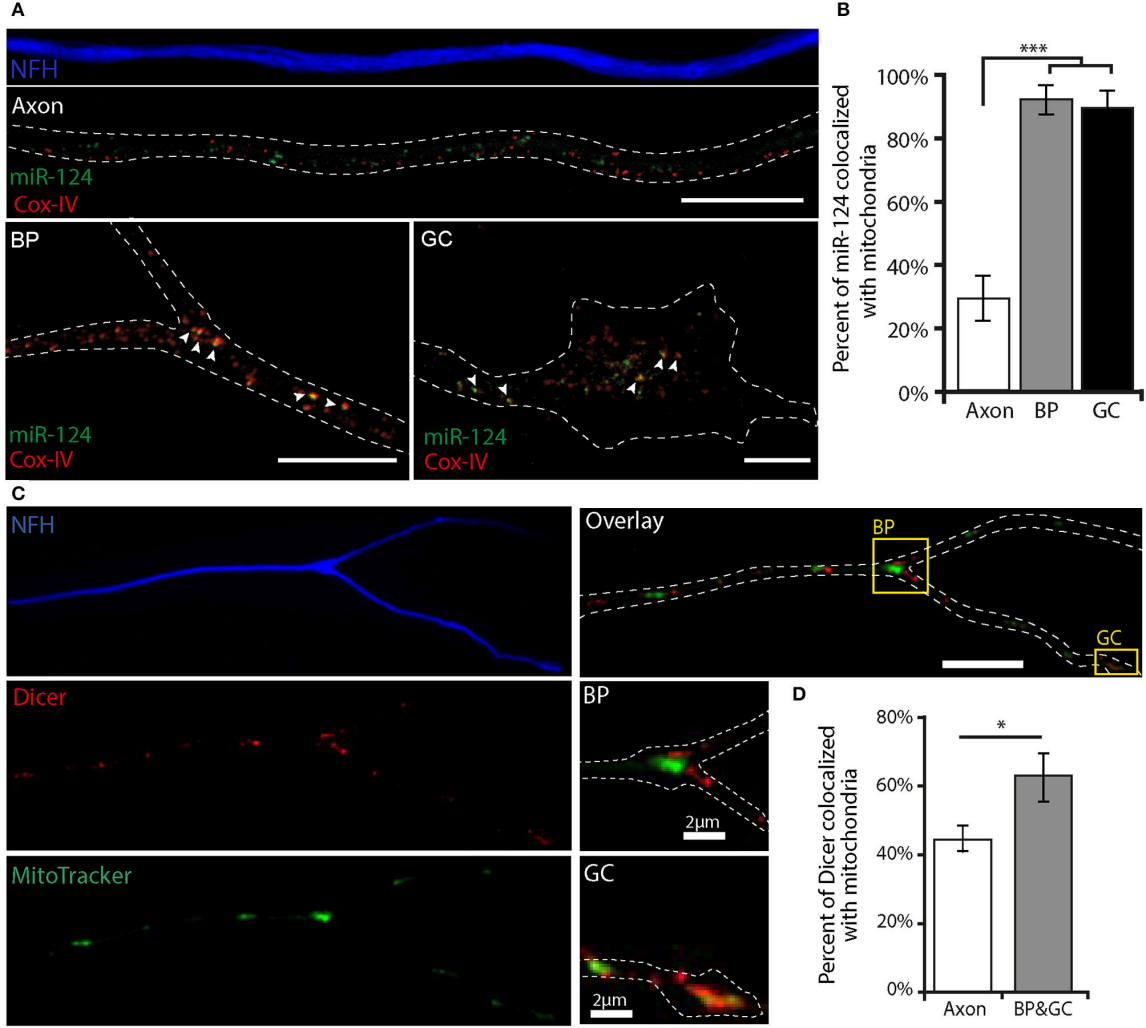
miR-124 and Dicer colocalize with mitochondria at axonal branch points and growth cones **(A)** *In situ* hybridization of miR-124, together with COX-IV protein to mark mitochondria in fixed MN culture, reveals a low rate of axonal colocalization in contrast to profound colocalization at axonal branch points (BP) and growth cones (GC). The white dashed line represents NFH borders; white arrowheads indicate colocalized puncta. **(B)** Quantitative analysis of miR-124 colocalization with COX-IV. ***p < 0.001 (*n* = 30). Scale bar = 10 μm. **(C)** Immunofluorescent images of MN stained for Dicer and NFH together with Mitotracker reveals a higher colocalization of Dicer with mitochondria in branch points and growth cones compared with axons. Scale bar = 10 μm. Insert scale bar = 2 μm. The white dashed line indicates NFH borders. **(D)** Quantitative analysis reveals a significantly higher colocalization of Dicer with mitochondria at growth cones and branch points *p < 0.05 (*n* = 11 axons).

Growing evidence suggests that stalled mitochondria correlate with sites of axonal branching and local mRNA translation. These suspended mitochondria generate “hot spots” for active translation by coordinating the localization of mitochondria, mRNA, and protein translation machinery (Spillane et al., 2013). Hence, we hypothesized that RNAi machinery also localizes to mitochondria at axonal branch points and growth cones. In order to test this, we plated primary MNs and performed *in-situ* hybridization for miR-124, and immunostaining for the mitochondrial protein Cox-IV (Figure 3A). We then analyzed the images for the colocalization coefficient of miR-124 and Cox-IV. A striking difference was evident upon comparison of the different neuronal compartments. Whereas, in the axonal shaft colocalization was weak (29 ± 7%), the colocalization of miR-124 with Cox-IV at axonal branch points and growth cones was significantly higher (91 ± 4% and 88 ± 6%, respectively, Figure 3B). To test whether this process also occurs with RISC proteins, MNs were immunostained for Dicer together with the mitochondrial dye Mitotracker (Figure 3C). During analysis, we distinguished between the axonal shaft and the growth cone/branch point. This analysis revealed that Dicer (channel A) to mitochondria (channel B) colocalization is enriched at growth cones and branch points (62 ± 7%) compared to the rest of the axon (43 ± 4%, Figure 3D) or random control (Supplementary Figure 3A). Control staining with TrkB antibody revealed only few colocalized puncta compared with mitochondria staining (Supplementary Figure 4). This finding suggests that mitochondria may play a role in the localization of RNAi machinery at specific sites along the axon.

### miR-124-Cy3 localizes to mitochondria at axonal branch points and growth cones

**Figure 4:**
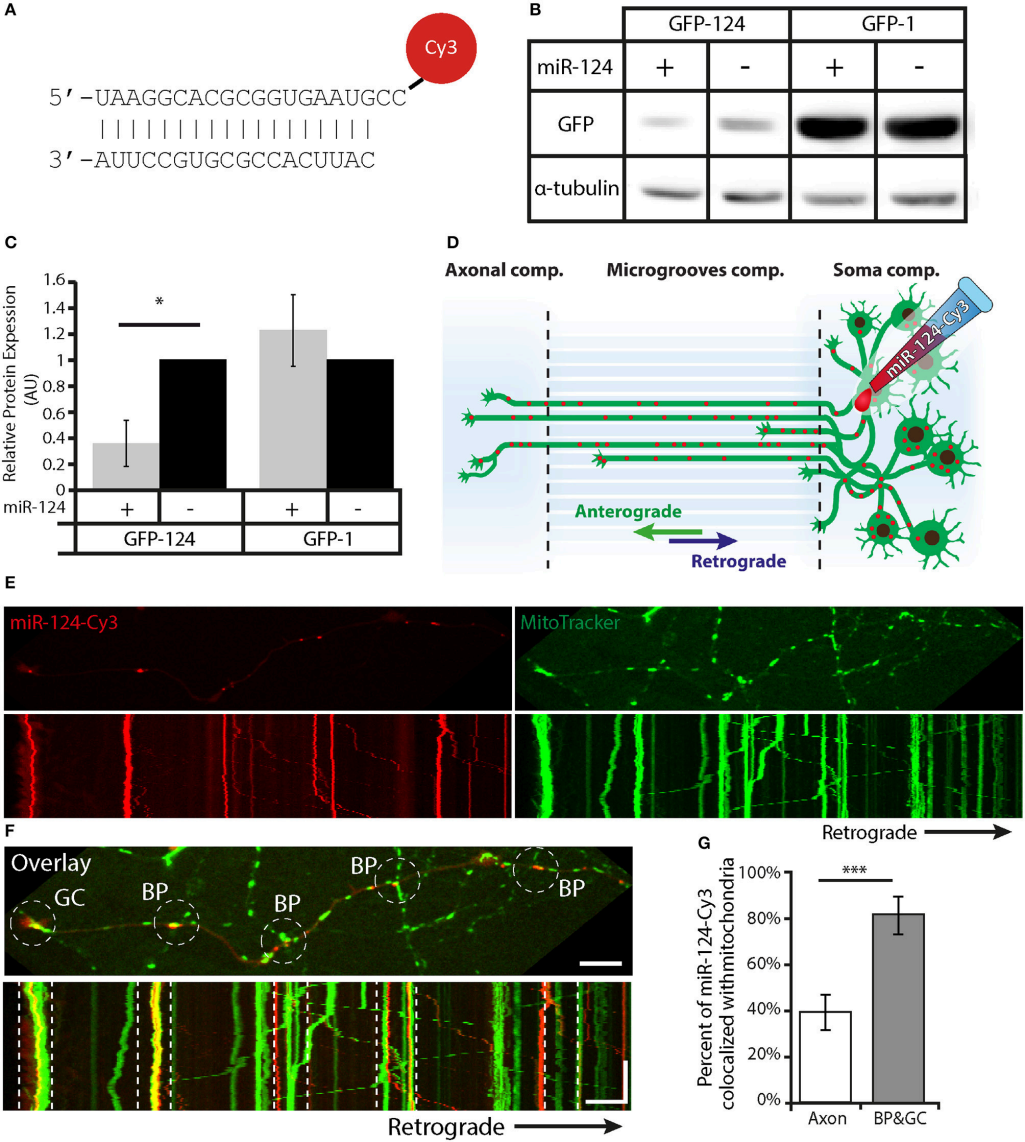
The functional miR-124-Cy3 construct colocalizes with mitochondria at axonal branch points and growth cones during live cell imaging. **(A)** Diagram of the miR-124-Cy3 construct. **(B)** Assay of miR-124-Cy3 functionality: representative western blot of GFP levels, with α-Tubulin as the loading control. **(C)** Quantification of GFP expression normalized to α-Tubulin shows that miR-124-Cy3 significantly and specifically represses GFP with a miR-124 recognition sequence. *p < 0.05. (*n* = 3 independent plates). **(D)** Diagram of the experimental platform: MNs were plated in the proximal side of a microfluidic chamber. Once the neurons extended their axons to the distal side (5–7 DIV), the proximal side was transfected with the miR-124-Cy3 construct. **(E)** Upper panel: Images from a time-lapse movie of miR-124-Cy3 (red) and Mitotracker (green) in distal axons. Lower panel: Kymographs obtained from movies of the same movie show displacement over time of both miR-124-Cy3 and mitochondria. Scale bar = 10 μm. **(F)** Overlay image and kymographs of **(E)** show the colocalization of miR-124-Cy3 with stalled mitochondria at axonal branch points and the growth cone. The white dashed circles and lines indicate the location of the branch point and growth cone in the upper and lower images, respectively. Scale bars: top = 10 μm, kymograph—Y-axis = 100 s, X-axis = 10 μm. **(G)** Quantitative analysis of kymographs reveals a higher colocalization of miR-124-Cy3 particles with mitochondria at growth cones and axonal branch points compared with axons. ***p < 0.001(*n* = 17 kymographs).

To study the transport and localization of miR-124 in live, developing neurons, we designed a synthetic construct similar to commercially available miRNA mimics, with the miR-124 sequence conjugated to a 3′ Cy3 tag (Figure 4A). The dissociation of the strands is an early occurrence, retaining the single-stranded mature miRNA structure with the conjugate florescent dye. To validate the functionality of miR-124-Cy3, we tested its ability to downregulate GFP levels in HEK293 cells, similarly to the assay described previously in Kaul et al. (2014). Briefly, cells were transfected with a GFP-expressing vector containing a miR-124 target site (GFP-124). As a control, a GFP-expressing vector with a miR-1 target site was used (GFP-1). The presence of miR-124-Cy3 reduced GFP-124 expression by approximately ~65 ± 17%, with no appreciable effect on GFP-1 expression (Figures 4B,C). Thus, miR-124-Cy3 is functional and specific.

To test the localization of miR-124-Cy3 in growth cones and branch points, we cultured MNs in microfluidic chambers, which separate the axonal and somatic compartments (Gluska et al., 2014; Zahavi et al., 2015). The neurons were grown for 5–7 days, and then miR-124-Cy3 was introduced into the somatic compartment (Figure 4D) together with Mitotracker. Live images of the distal compartment were collected every 3 s for 100 frames. Kymographs generated from these images were analyzed for overlapping tracks between the different channels (Figures 4E,F; Supplementary Movie 1). These revealed a significant increase in the colocalization of miR-124-Cy3 (channel A) to mitochondria (channel B) tracks specifically at growth cones and branch points (39.4 ± 7.5% of total miR-124-Cy3 colocalized with mitochondria in axons compared with 81.8 ± 7.6% in growth cones and axonal branch points; Figure 4G and random analysis in Supplementary Figure 5A).

### mir-124-Cy3 is transported in acidic compartments along axons

**Figure 5:**
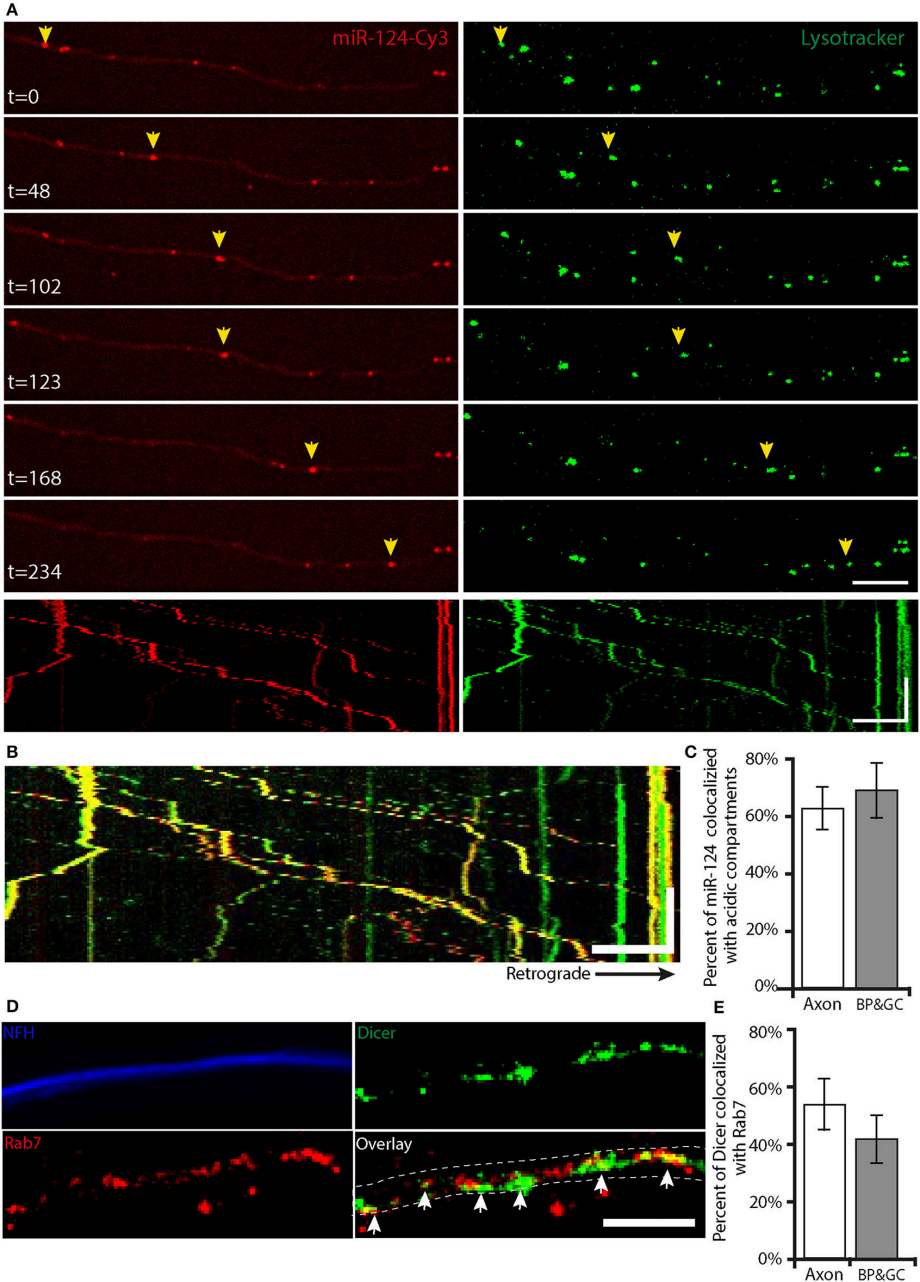
RNAi components are transported with acidic compartments along the axon **(A)** Time lapse images of motor neuron axons show the axonal transport of miR-124-Cy3 (red) and acidic compartments (green; Lysotracker). Scale bar = 10 μm. Yellow arrows indicate the transport of a single particle over time. The lower panel shows kymographs of these movies. Scale bars: X-axis = 10 μm, Y-axis = 100 s. **(B)** Overlay kymographs show that the majority of the transported miR-124-Cy3 particles colocalize with acidic compartments. **(C)** Analysis and quantification of kymographs reveal no difference in the colocalization of miR-124-Cy3 particles with acidic compartments at growth cones and axonal branch points compared to axons (*n* = 17 kymographs). **(D)** Immunofluorescent images of primary MN stained for Dicer (green), Rab7 (red), and NFH (blue) demonstrate the colocalization of Dicer and endosomal marker in axons. Scale bar = 10 um. The dashed line indicates the borders of NFH; white arrows indicate the colocalized particles. **(E)** Colocalization analysis reveals no significant difference in the colocalization of Dicer with Rab7 in branch points and growth cones compared to axons (*n* = 11 axons).

To complete our model of axonal RNAi localization, we sought to decipher the transport mechanism by which microRNA travel to and from distal axonal compartments. Acidic compartments have previously been shown to play a role in the transport and localization of RISC (Siomi and Siomi, 2009); thus, we added the acidic compartment dye Lysotracker to the neurons prior to imaging. Time-lapse imaging of axons in the groove compartment was performed, and particle tracking was analyzed to reveal a high level of concordance between transported miR-124-Cy3 and acidic compartments kymographs (Figures 5A,B; Supplementary Movie 2). Analysis of overlapping tracks in kymographs from the distal and axonal groove compartments revealed that transporting miR-124-Cy3 (channel A) colocalized extensively with acidic compartments (channel B), (62.2 ± 7.6%). Interestingly, there was no significant enrichment of this colocalization at the axonal branch points and growth cones (69.3 ± 8.7%; Figure 5C; Supplementary Figure 5B). Since several different organelles fall under the description of acidic compartments, we investigated whether the RISC complex specifically colocalizes in axons with the endosomal marker Rab7 (Figure 5D). Axonal analysis demonstrates a colocalization coefficient of 53 ± 8% between Dicer (channel A) and Rab7 (channel B; Figure 5E; Supplementary Figure 3B) in axons, and 41 ± 8% in growth cones and branch points, hence there is no preferential colocalization of Dicer and Rab7 in the vicinity of growth cones and branch points, which is comparable to the previously observed colocalization of acidic compartments and miR-124-Cy3.

### miR-124-Cy3 has increased retrograde motility and decreased mitochondrial localization in mSOD1 neurons

Mitochondrial damage and alterations in protein synthesis are hallmarks of motor neuron toxicity (Kapur et al., 2017; Vandoorne et al., 2018). In order to investigate possible alterations in RNAi levels and localization in ALS neurons, we tested the levels of Dicer and Ago2 in neuronal tissues from mSOD1 compared to WT littermate controls. Although Dicer levels in sciatic axoplasm were highly variable, we could detect no significant difference in their levels (Supplementary Figure 6). However, Dicer localization to mitochondria was significantly decreased in mSOD1 axons (Supplementary Figures 7A,B, 50 ± 3% and 34 ± 3% in WT neurons and mSOD1 neurons, respectively), while colocalization with Rab7 was not altered (Supplementary Figures 7A,C, 51 ± 6% and 46 ± 4% in WT and mSOD1 neurons, respectively).

**Figure 6:**
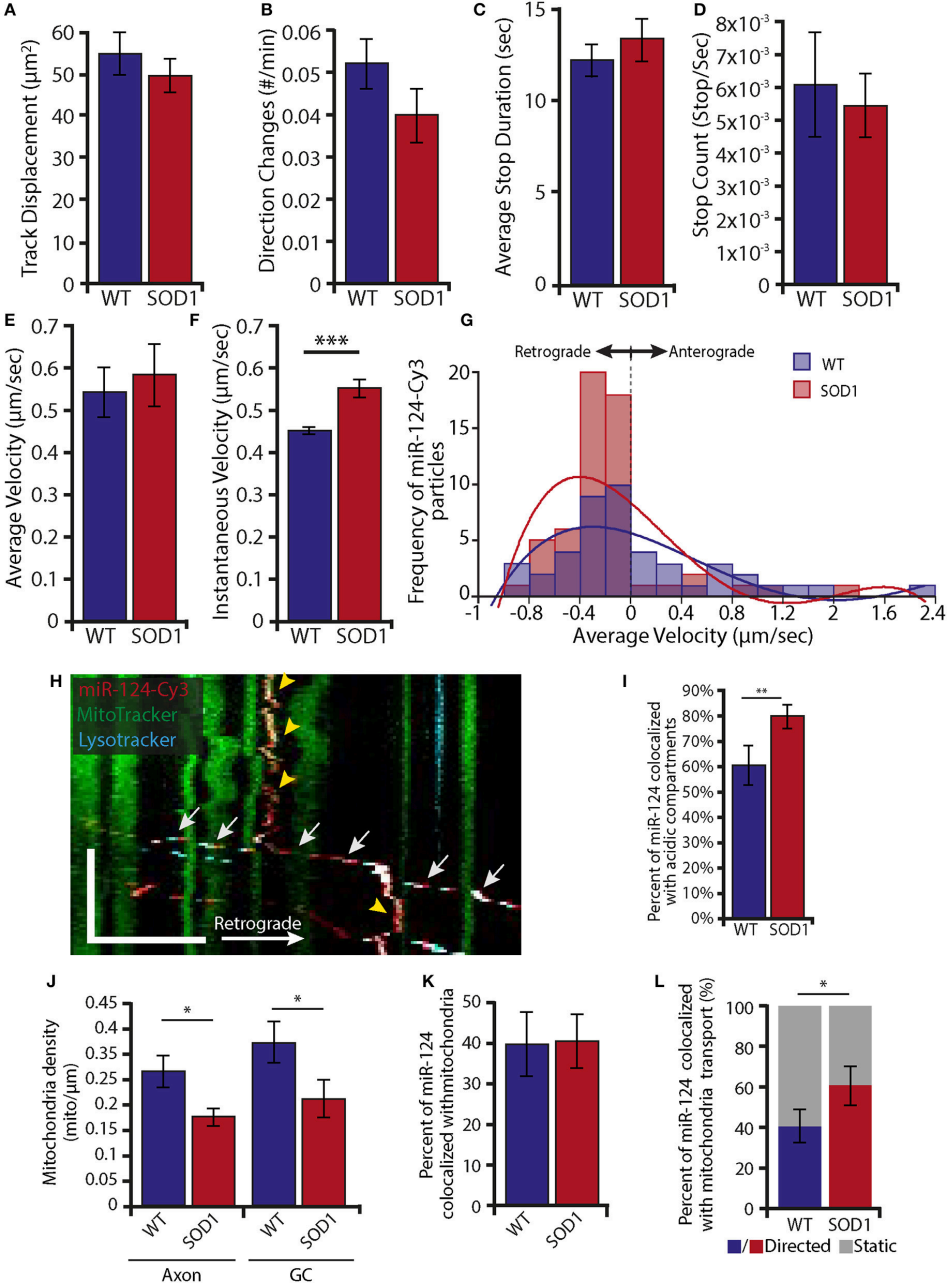
Increase in retrograde transport of miR-124-Cy3 together with reduced localization to static mitochondria in mSOD1 MNs **(A–E)** Single particle tracking analyses of miR-124-Cy3 axonal transport in WT and mSOD1 MN indicate no alterations in the following parameters: track displacement **(A)**, number of direction changes **(B)**, average stop duration **(C)**, stop count **(D)**, and average velocity **(E)**. **(F)** The instantaneous velocity of miR-124-Cy3 particles was higher in mSOD1 MN (***p < 0.001). **(G)** Distribution plots of miR-124-Cy3 tracks by average velocity indicate that a higher proportion of miR-124-Cy3 is transported retrogradely at lower velocities (Binning 0.2 μm/s; For A-G: *n* of miR-124-Cy3 particles: WT = 42; SOD1 = 59). **(H)** Representative axonal kymograph displays miR-124-Cy3 transports together with acidic compartments and stops at sites of stationary mitochondria. Scale bars: Y-axis = 100 s, X-axis = 10 μm. The white arrows indicate a transporting acidic compartment with miR-124-Cy3; the yellow arrowheads indicate stationary miRNA with mitochondria. **(I)** Kymograph analysis reveals a higher axonal colocalization of miR-124-Cy3 particles with acidic compartments in mSOD1 (**p < 0.01). **(J)** Time lapse movies from WT and mSOD1 MN indicate a decrease in mitochondrial density under ALS conditions, both in axons (left panel) and growth cones (GC, right panel). (*p < 0.05), **(K)** Kymograph analysis reveals a similar axonal colocalization of miR-124-Cy3 particles with mitochondria in WT and mSOD1 mutated MN.). **(L)** Kymograph analysis reveals increased colocalized transport of miR-124-Cy3 with axonal mitochondria in mSOD1 MN compared to WT. (*p < 0.05; WT: *n* = 17, mSOD1: *n* = 23). For H-L: 135 and 119 miR-124-Cy3 and 464 and 449 mitochondria tracks were analyzed in a total of 17 WT and 23 SOD1 kymographs, respectively.

Since we cannot rule out dynamic changes that lead to Dicer mislocalization, we tested for alterations in miRNA. To this end, we collected live cell images of miR-124-Cy3 in both WT and mSOD1 neurons cultured in microfluidic chambers. Transport parameters of miR-124-Cy3, including track displacement, direction changes, average stop duration, stop count, average velocity, and instantaneous velocity were analyzed (Figures 6A–G). We observed a small but significant increase (~10%) in the instantaneous velocity between WT (0.45 ± 0.01 μm/s) and mSOD1 (0.55 ± 0.02 μm/s; Figure 6F). Interestingly, this increase was limited to retrograde transporting particles (Figure 6G), indicating increased retrograde transport of miR-124-Cy3 particles. We thus continued to investigate whether the localization of miR-124-Cy3 particles to acidic compartments and mitochondria is altered in ALS MNs, using a double stain of Lysotracker together with Mitotracker (Figure 6H). Analysis of the colocalized tracks revealed significant differences in the percentage of axonal mir-124-Cy3 particles that colocalized with acidic compartments (62 ± 7.6% localization in WT axons, compared to 82 ± 5.1% in mSOD1), which together with the increased retrograde transport, suggest a decreased static localization of RNAi (Figure 6I). To test if the increased miR-124-Cy3 motility is associated with mitochondrial alterations, we quantified axonal mitochondria density (number of mitochondria per micron) in WT and mSOD1 axons. In line with previous work (Wiedemann et al., 2002), we observed a decrease in mitochondria density both in axonal compartment (0.29 ± 0.03 in WT compared to 0.19 ± 0.01 in mSOD1), and in the growth cone (0.35 ± 0.05 in WT compared to 0.23 ± 0.04 in mSOD1, Figure 6J). However, the percent of colocalized miR-124-Cy3 to mitochondria remained similar (39.14 ± 7.5% localization in WT axons, compared to 40.15 ± 6.5% in mSOD1, Figure 6K), indicating no clear dissociation of miR from the mitochondria. We then tested if mitochondrially-localized miR-124-Cy3 particles distribute differently between transporting and static mitochondria. In WT axons, 59.62 ± 8.3% of mitochondria-localized miR-124-Cy3 particles were static, while in SOD axons 37.1 ± 9.19% of localized particles were static (Figure 6L). Taken together, this suggests that miR-124-Cy3 has increased retrograde transport in ALS neurons, which may be facilitated both by mitochondria evacuation and acidic compartment association. The possibility that mitochondria serves as a stress-induced carrier for miRNAs is an intriguing avenue for future research.

**Figure 7:**
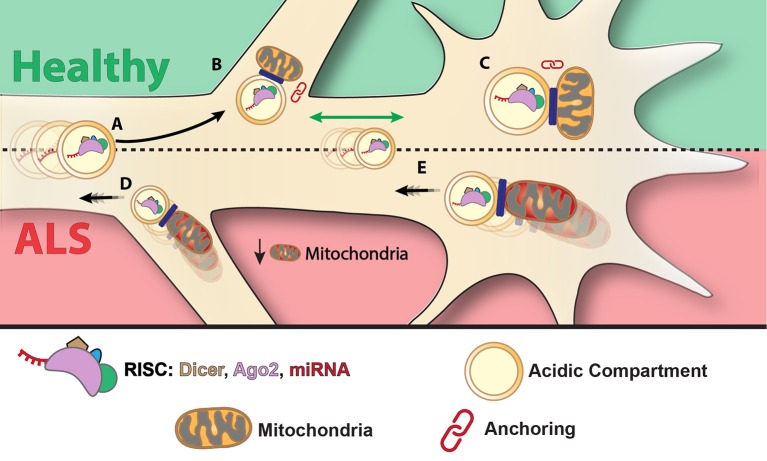
Suggested model: mitochondria anchors RISC at axonal branch points and growth cones—a mechanism that is altered in ALS. miRNAs are transported in acidic compartments, possibly together with RISC **(A)**. miRNA and RISC components associate with mitochondria and anchor to axonal branch points **(B)** and growth cones **(C)**. Under pathological conditions such as in ALS, the RNAi-mitochondria complex may be released from axonal branch points **(D)** and synapses **(E)** and transported retrogradely to the cell body.

Altogether, our data may suggest a model in which RNAi localizes with stalled mitochondria in “hot spots” of local synthesis such as axonal branch points and growth cones, and that RNAi is transported in acidic compartments such as Rab7 endosomes (Figure 7). In ALS MNs, there is an increase in retrograde transport of miR-124 particles, and higher localization of those to acidic compartments and to moving mitochondria, possibly suggesting that RNAi machinery is cleared away from the diseased axon along with these organelles. A possible underlying cause for this process may be disease-related disruption to the mitochondria. Additional research may shed light on the relevance of this process to ALS, as well as other neurodegenerative disorders.

## Discussion

In this study, we have demonstrated that the silencing machinery components, namely, Dicer, Ago2, and miRNAs, are localized in axons far from the perinuclear region and can be associated with mitochondria. This association is increased at sites where local translation in axons was shown to occur. Axonal transport assays of a synthetic mimic of the neuronally enriched miR-124 revealed that this miRNA colocalizes with transporting acidic compartments, and with static mitochondria. An intriguing possibility is that miRNA transports with acidic compartments and halts with stalled mitochondria. Moreover, it is highly probable that additional neuronal miRNAs employ this mechanism. Furthermore, we have shown that the transported miR-124 localized to mitochondria is affected by the ALS-associated mutation mSOD1. Taken together, these findings may shed light on how the silencing machinery localizes in axons, describe a putative novel role for axonal mitochondria, and identify a mechanism that allows regulation of local synthesis.

### RISC in the axon

The large number of mRNA transcripts discovered in both dendrites and axons suggests that local protein translation is the rule and not the exception (tom Dieck et al., 2014; Rotem et al., 2017; Zappulo et al., 2017). Although it is not fully understood how mRNA transcripts localize to distinct neuronal compartments, the majority of mRNA is translationally repressed in the axon, waiting for an extrinsic cue (i.e., stress, trophic factors, or injury). This repression is also crucial during axonal transport, and recent work suggests that miRNAs, which are transported together with target mRNAs, contribute to this silencing (Peredo et al., 2014).

Both pre- and mature miRNAs have been identified in distal parts of the neuron (Briese et al., 2015; Vargas et al., 2016). In dendrites, miRNAs have been shown to undergo Dicer-dependent maturation in a precise location in response to external stimuli (Sambandan et al., 2017). In axons, pre-miRNAs were found to fluctuate in response to injury in distal parts (Kim et al., 2015; Vargas et al., 2016). Together, this points to an active miRNA maturation mechanism in the axon. Our results, together with previous studies, suggest that the association of Dicer with pre- and mature miRNAs and with mitochondria may play a role in mitochondrial function.

### miRNA axonal transport in acidic compartments

A major challenge in this study was to establish a system to view miRNA transport in live cells. The localization of the miRNA-Cy3 construct we used was to a large extent with the acidic compartment marker LysoTracker. Acidic compartments serve various functions in the cell, and include early and late endosomes, lysosomes, portions of the trans-golgi membrane, and certain secretory vesicles (Anderson and Orci, 1988). The endosomal pathway is a highly conserved, essential pathway that is used by the cell to import and then sort extracellular molecules. In neurons in particular, endosomes take on an additional signaling role. Their rapid retrograde transport serves as an efficient mechanism for signal transduction across large distances (i.e., neurotrophin transport from the distal synapse to the cell body; Howe and Mobley, 2004), or a mechanism to regulate the nature and intensity of a signal (Zahavi et al., 2018). A link between endosomal trafficking and RNA silencing was first established when the RISC components Ago2 and GW182 were found to be enriched in their fraction of multivesicular bodies (MVBs), suggesting that these foci are distinct from P-bodies (Gibbings et al., 2009; Siomi and Siomi, 2009). Moreover, this raises the possibility that MVBs can constitute sites of RISC assembly and/or function, a suggestion supported by identifying miRNA-repressible mRNAs in MVB fractions (Gibbings et al., 2009). Furthermore, the blocking of MVB formation and maturation compromises miRNA function (Gibbings et al., 2009; Lee et al., 2009; Siomi and Siomi, 2009). Taken together, these studies suggest several possible roles for MVBs in regulating RISC activity. Our observations support the possibility that the endosomal pathway transports miRNAs from the cell periphery to the cell body, as well as to or from sites of local translation, as needed by the cell.

Previous work that introduced siRNA into distal axons concluded that it acts locally to regulate translation, and reported minimal retrograde diffusion of the fluorescently marked siRNA (Hengst, 2006). Our work differs in multiple technical aspects, which may account for the differences between our findings and the previous results: (1) our method of introduction differed; (2) we worked on a different cell type; (3) importantly, our construct was designed as a mimic for an endogenous miRNA, that was introduced as a double stranded entity to ensure stability, as opposed to a single-stranded siRNA. These differences make a compelling case for further investigation into the nature of miRNA transport in different cell types, maturation levels and under various conditions.

### miR-124-mitochondria colocalization at translational “hotspots”

We observed that Dicer puncta appear to aggregate along the axon in distinct locations such as the growth cone and branch points where local translation is necessary (Cioni et al., 2018). Notably, these sites were previously shown to be enriched with mitochondria and translational machinery, creating a spatiotemporally coordinated translational “hotspot” (Spillane et al., 2013; Wong et al., 2017).

The mitochondria and translation machinery may be solely colocalized to provide the energetic requirements of local protein synthesis; however, it has become apparent in recent years that mitochondria are necessary for an additional localizing function, such as serving as a signaling hub, a scaffold for translational activity, or perhaps play a transporting role for its various components (Spillane et al., 2013; Su et al., 2014). These questions will be the key for future investigation.

Our data suggest that in addition to all of these functions, mitochondria can serve also as an anchor for localizing regulatory RNAi determinants such as Dicer and miRNAs. This further supports recent discoveries of miRNA in axonal mitochondria (Vargas et al., 2016). The association we show between Dicer, miR-124, and mitochondria also suggests a role for mitochondria in the regulation of local translation by RNAi, as well as a role for specific miRNAs in regulating the synthesis of mitochondrial proteins.“mitomiRs,” as mitochondria-associated miRNAs have been named, include, among others, nuclear-encoded miRNAs acting in the nucleus or cytoplasm, and nuclear-encoded miRNAs acting at mitochondria (Bandiera et al., 2013).

### Disrupted RNAi localization in ALS

MNs are the primary target for neurodegeneration in ALS. These neurons have high energetic needs, but limited energy stores. They are therefore highly dependent on continuous energy provision, which makes them especially vulnerable to energetic shortages (Le Masson et al., 2014; Cestra et al., 2017). Several levels of mitochondrial dysfunction have been described in ALS, including defects in morphology, distribution, and function. Since SOD1 has been found to regulate ROS in the cytoplasm, and to associate with the inter-membranal space in the mitochondria of neurons, mitochondrial dysfunction is highly likely to be one of the mechanisms by which mutations in SOD1 can lead to neurodegeneration. Indeed, mutated SOD1 has been shown to impair both mitochondrial number, function and motility, possibly as a result of aggregation, prior to symptom onset (Edens et al., 2016).

Our work describes a decrease in the levels of mitochondria, and in the levels of association between Dicer and mitochondria, together with increased retrograde transport of the miR-124 mimic, and its increased association with acidic compartments and transporting mitochondria. Taken together, these point to a depletion of RNAi machinery in the diseased axon, and to mitochondria and acidic compartments, mostly endosomes, as a putative mechanism for facilitating this clearance. From a functional point of view, this depletion may contribute to the aberrant local protein synthesis observed in neurodegenerative diseases.

The association we describe between mitochondria, Dicer and miR-124 in the mSOD1 ALS model suggests that this association is essential for fine-tuning local protein synthesis in the axon, an ability that may be lost in the early stages of the disease, thus contributing to the progressive nature of axonal degeneration and further strengthening the importance of local protein synthesis to synapse maintenance and axonal stability in ALS.

## Author contributions

NG-E, TA and EP: conceptualization; NG-E, TA, AI, CC, TG-P, DW, and EP: methodology; NG-E, TA, AI, and TG-P: validation; NG-E, TA, CC, and TG-P: formal analysis; NG-E, TA, AI, CC, and TG-P: investigation; DW and EP: resources; NG-E, TA, and EP: writing; NG-E, TA, AI, CC, TG-P, DW, and EP: visualization; DW and EP: supervision.

### Conflict of interest statement

The authors declare that the research was conducted in the absence of any commercial or financial relationships that could be construed as a potential conflict of interest.

## Supplementary material

Supplementary Movie 1 miR-124-Cy3 colocalizes with stationary mitochondria in growth cones: live-cell imaging of miR-124-Cy3 particles (red) together with mitotracker marked mitochondria (green) in a motor neuron growth cone. The full arrow shows stationary miR-124-Cy3 with mitochondria, 12 fps.

Supplementary Movie 2 miR-124-Cy3 colocalizes with moving acidic compartment in axons: live-cell imaging of miR-124-Cy3 particles (red) together with the lysotracker marked acidic compartment (green) in a motor neuron axon. The full arrow shows moving miR-124-Cy3 with an acidic compartment, 12 fps.

Supplementary Figure 1 Dicer and Ago2 antibody staining is specific: Dicer **(A)** and Ago2 **(B)** can be detected in a punctate pattern in the axonal growth cone of fixed MNs (green) while the axon is detected by HB9 (Blue). Control with secondary antibody only, detected no fluorescent signal **(C)**. Scale bar = 10 μm.

Supplementary Figure 2 RISC localizes to the NMJ: **(A)** Dicer and Ago2 NMJ staining is specific, as can be visualized by lack of fluoresence in secondary only panel. **(B–E)** Images of both 40× **(B,C)** and 100× magnification **(D,E)** reveal Dicer and Ago2 enrichment in the NMJ region, including both the pre-synaptic axonal side (visualized by NFH) and post-synaptic muscular side (visualized by BTX). Co-localization analysis indicates Dicer and Ago2 colocalization with NFH stain. Scale bar = 10 μm.

Supplementary Figure 3 Colocalization analysis of Dicer with mitochondria and Rab7 is not random: **(A,B)** Immunofluorescent images of MN stained for Dicer and NFH together with Mitotracker to test colocalization of Dicer with mitochondria **(A)** and Rab7 **(B)**. Quantitative analysis after randomizing the colocalization by flipping axon direction (random) resulted in a significantly decreased colocalization coefficient compared to original images (true). ^*^*p* < 0.05 (*n* = 6 axons) for **(A)**. ^**^*p* < 0.01 (*n* = 6 axons) for **(B)**.

Supplementary Figure 4 Dicer colocalizes with mitochondria but not with TrkB: immunofluorescent images of HB9 marked MN growth cone stained for Dicer reveals few colocalized puncta with TrkB **(A)** compared to Mitotracker colocalization **(B)**. Dashed line indicates HB9 axonal borders. Yellow arrows indicate colocalized puncta. Scale bar = 10 μm.

Supplementary Figure 5 Colocalization analysis of kymographs is not random: **(A,B)** Analysis of analyzed kymographs to test colocalization of miR-124-Cy3 with mitotracker **(A)** and lysotracker **(B)** under WT (left panel) or mSOD1 mutation (right panel) reveals that flipping kymograph direction resulted in a profound decrease in colocalization ratios. ^**^*p* < 0.01 (*n* = 17 kymographs for WT, *n* = 26 kymographs for SOD1) for **(A)**. ^***^*p* < 0.001 (*n* = 17 kymographs for WT, *n* = 26 kymographs for SOD1) for **(B)**.

Supplementary Figure 6 SOD1 mutation causes no change in levels of RISC in mice tissues: **(A,B)** Western blot of extracts from five p120 mSOD1 and five littermate (LM) mouse spinal cords **(A)** and sciatic nerves **(B)** immunoblotted for Dicer and Ago2; gERK is used as a loading control. **(C,D)** Quantification of protein levels in spinal cord (*n* = 5) reveals no change in Dicer **(C)** and Ago2 **(D)** protein levels. **(E,F)** Quantification of protein levels in sciatic nerve (*n* = 5) reveals highly variable levels of Dicer **(E)** and no change in Ago2 **(F)** protein levels.

Supplementary Figure 7 SOD1 mutation causes reduced Dicer and mitochondria colocalization in MN: **(A)** Immunofluorescent images of primary motor neuron axons stained for Dicer (red), mitochondria (green), Rab7 (magenta). Overlay images show no difference in Rab7 and Dicer colocalization between WT and mSOD1 axons. Dicer colocalization with mitochondria is reduced in the growth cone (GC) in mSOD1 axons. The white dashed line indicates growth cone borders. **(B,C)** Colocalization analysis reveals a decreased colocalization of Dicer and mitochondria in mSOD1 neurons **(B)** but a similar colocalization of Dicer with Rab7 (**C**; ^**^*p* < 0.01, *n* = 11 axons for WT, *n* = 11 axons for SOD1). Scale bar = 10 μm.
