# Supplementary figures and images for: Localization of RNAi Machinery to Axonal Branch Points and Growth Cones Is Facilitated by Mitochondria and Is Disrupted in ALS
Source: Front Mol Neurosci. 2018 Sep 5;11:311. doi: 10.3389/fnmol.2018.00311 (PMC6134038; doi:10.3389/fnmol.2018.00311)

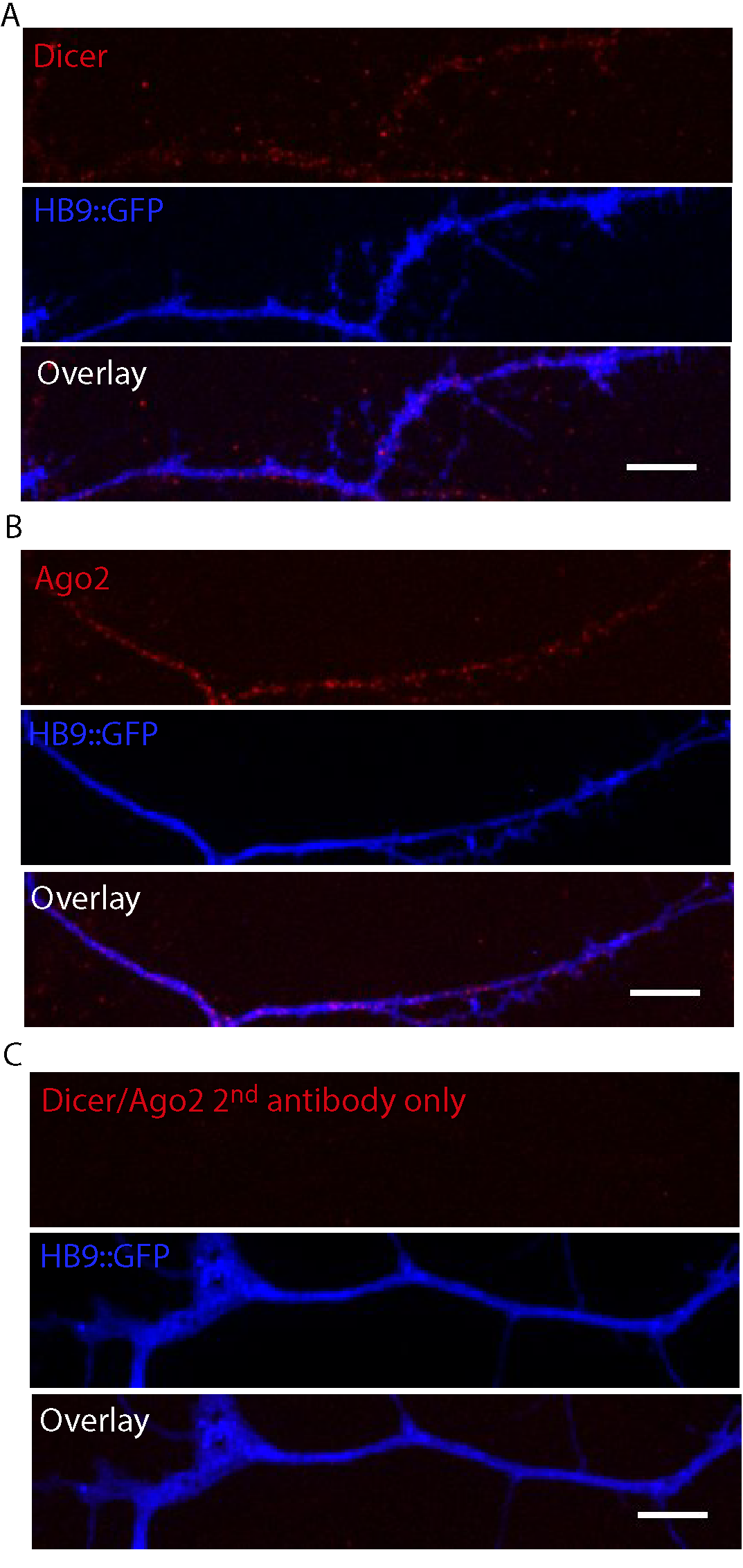

Supplement: Supplementary Figure 1 — Dicer and Ago2 antibody staining is specific: Dicer (A) and Ago2 (B) can be detected in a punctate pattern in the axonal growth cone of fixed MNs (green) while the axon is detected by HB9 (Blue). Control with secondary antibody only, detected no fluorescent signal (C). Scale bar = 10 μm. [file Image_1.TIF]

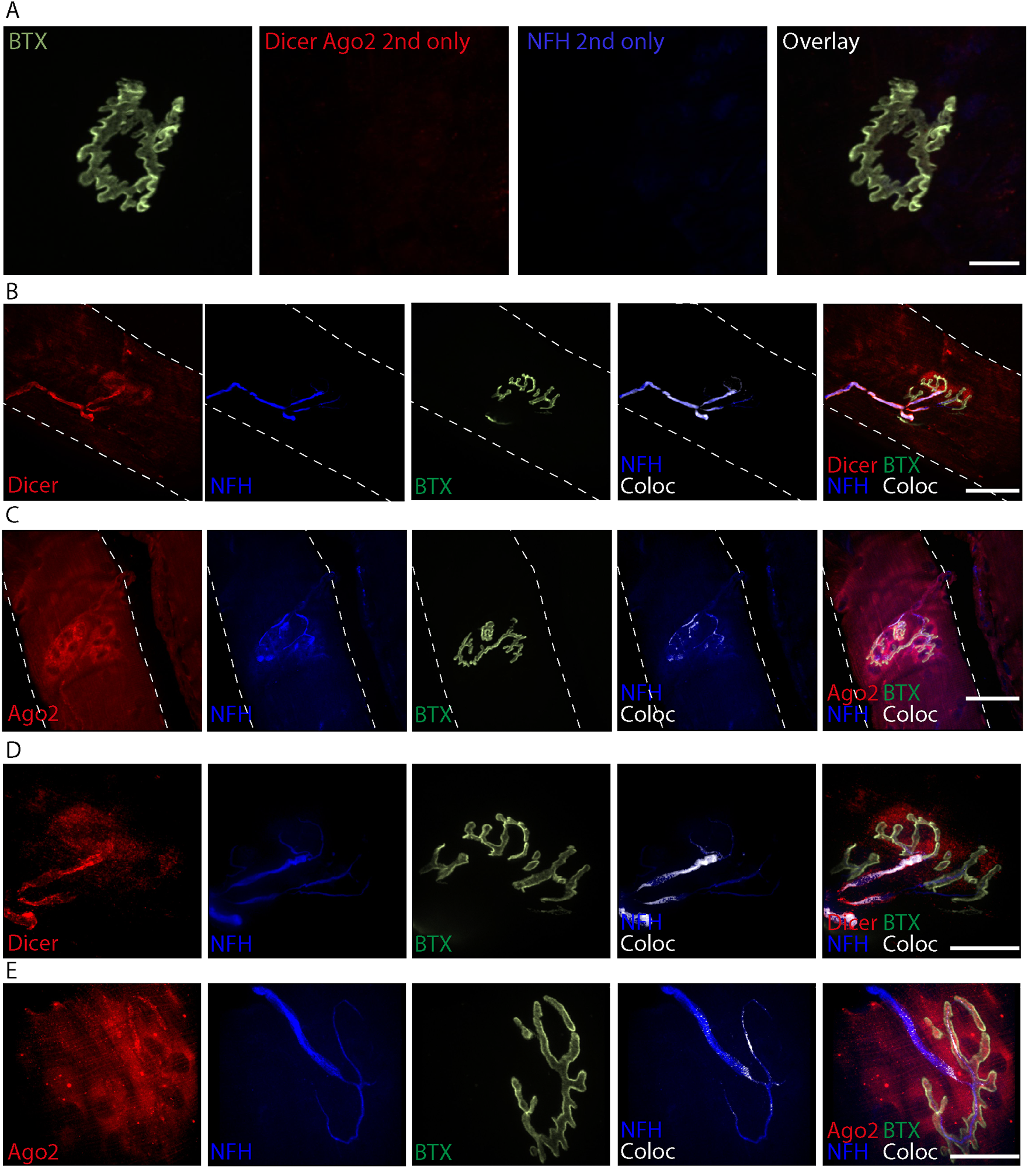

Supplement: Supplementary Figure 2 — RISC localizes to the NMJ: (A) Dicer and Ago2 NMJ staining is specific, as can be visualized by lack of fluoresence in secondary only panel. (B–E) Images of both 40× (B,C) and 100× magnification (D,E) reveal Dicer and Ago2 enrichment in the NMJ region, including both the pre-synaptic axonal side (visualized by NFH) and post-synaptic muscular side (visualized by BTX). Co-localization analysis indicates Dicer and Ago2 colocalization with NFH stain. Scale bar = 10 μm. [file Image_2.TIF]

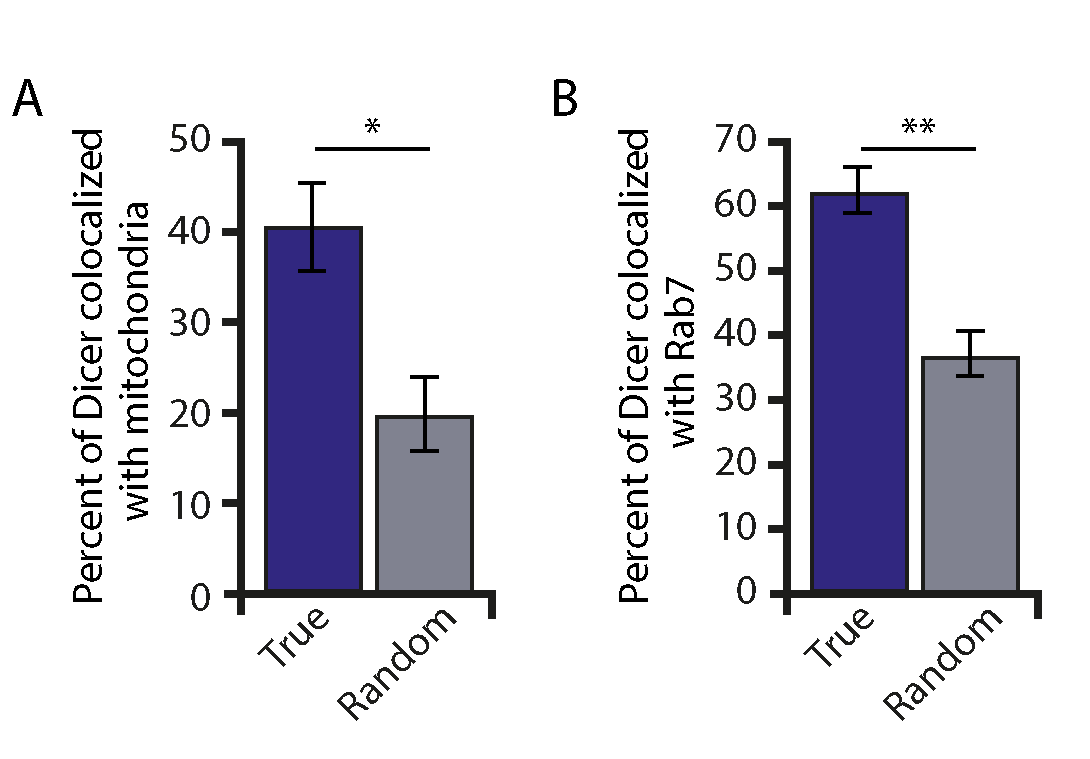

Supplement: Supplementary Figure 3 — Colocalization analysis of Dicer with mitochondria and Rab7 is not random: (A,B) Immunofluorescent images of MN stained for Dicer and NFH together with Mitotracker to test colocalization of Dicer with mitochondria (A) and Rab7 (B). Quantitative analysis after randomizing the colocalization by flipping axon direction (random) resulted in a significantly decreased colocalization coefficient compared to original images (true). *p < 0.05 (n = 6 axons) for (A). **p < 0.01 (n = 6 axons) for (B). [file Image_3.TIF]

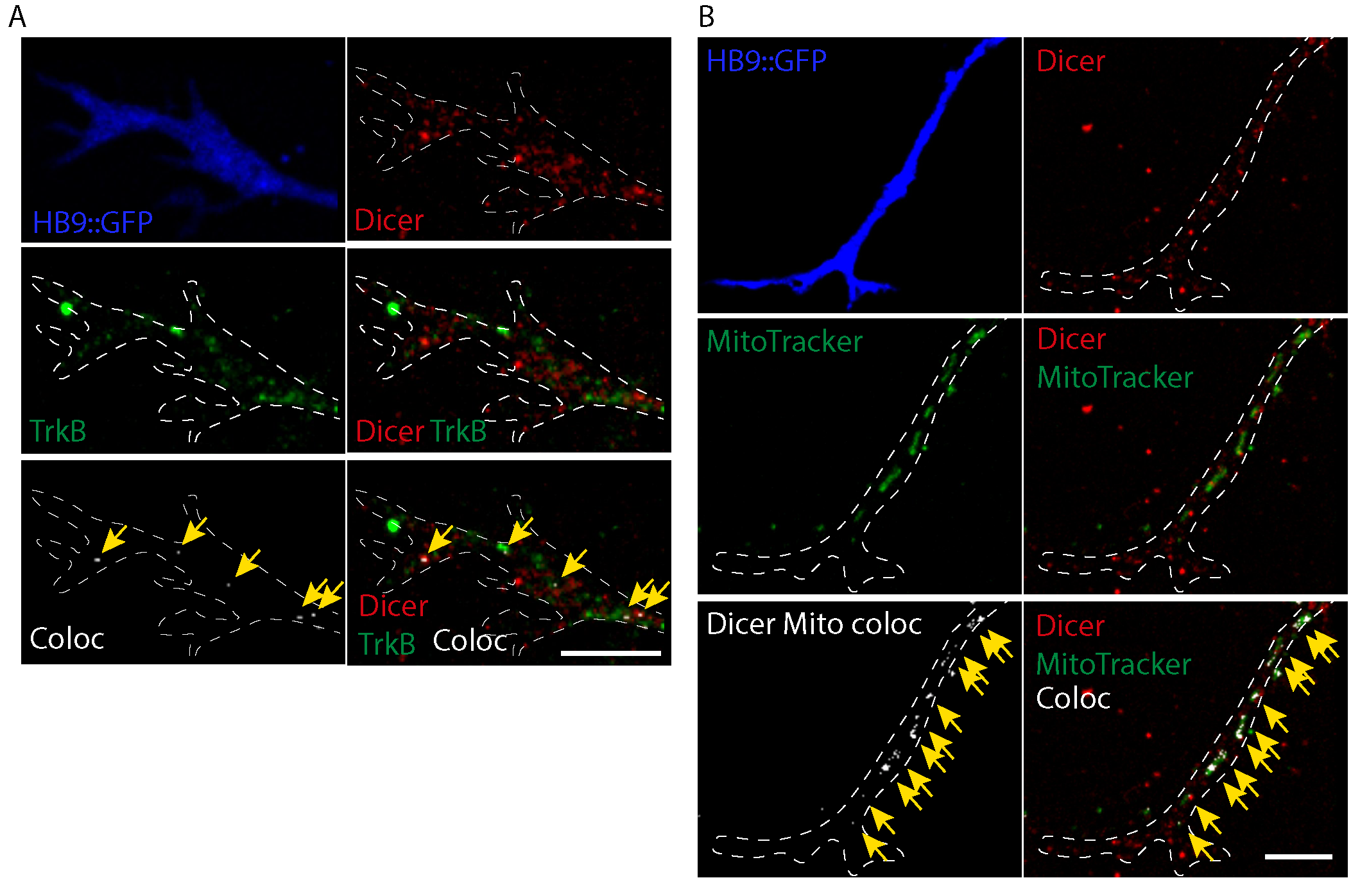

Supplement: Supplementary Figure 4 — Dicer colocalizes with mitochondria but not with TrkB: immunofluorescent images of HB9 marked MN growth cone stained for Dicer reveals few colocalized puncta with TrkB (A) compared to Mitotracker colocalization (B). Dashed line indicates HB9 axonal borders. Yellow arrows indicate colocalized puncta. Scale bar = 10 μm. [file Image_4.TIF]

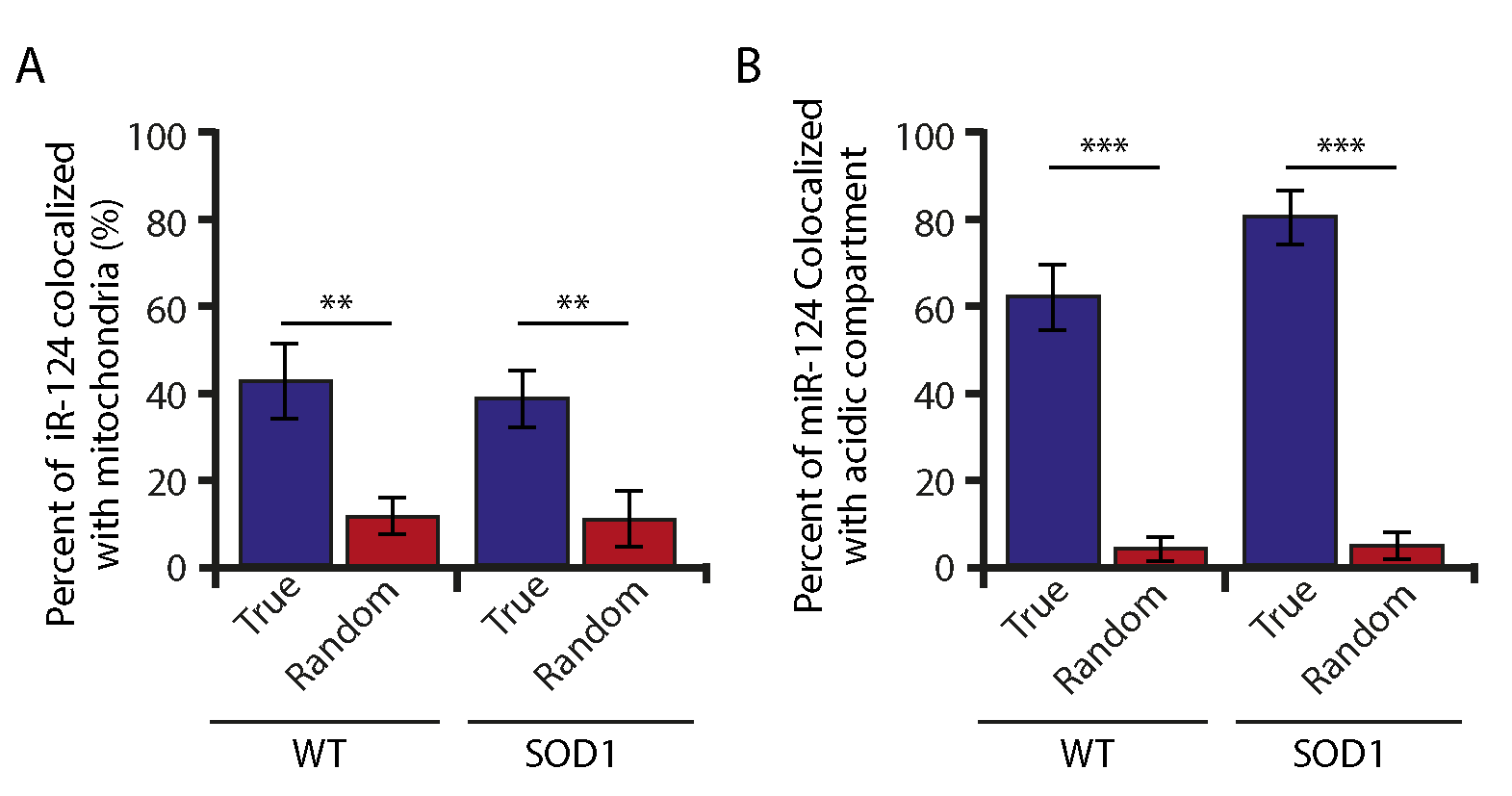

Supplement: Supplementary Figure 5 — Colocalization analysis of kymographs is not random: (A,B) Analysis of analyzed kymographs to test colocalization of miR-124-Cy3 with mitotracker (A) and lysotracker (B) under WT (left panel) or mSOD1 mutation (right panel) reveals that flipping kymograph direction resulted in a profound decrease in colocalization ratios. **p < 0.01 (n = 17 kymographs for WT, n = 26 kymographs for SOD1) for (A). ***p < 0.001 (n = 17 kymographs for WT, n = 26 kymographs for SOD1) for (B). [file Image_5.tif]

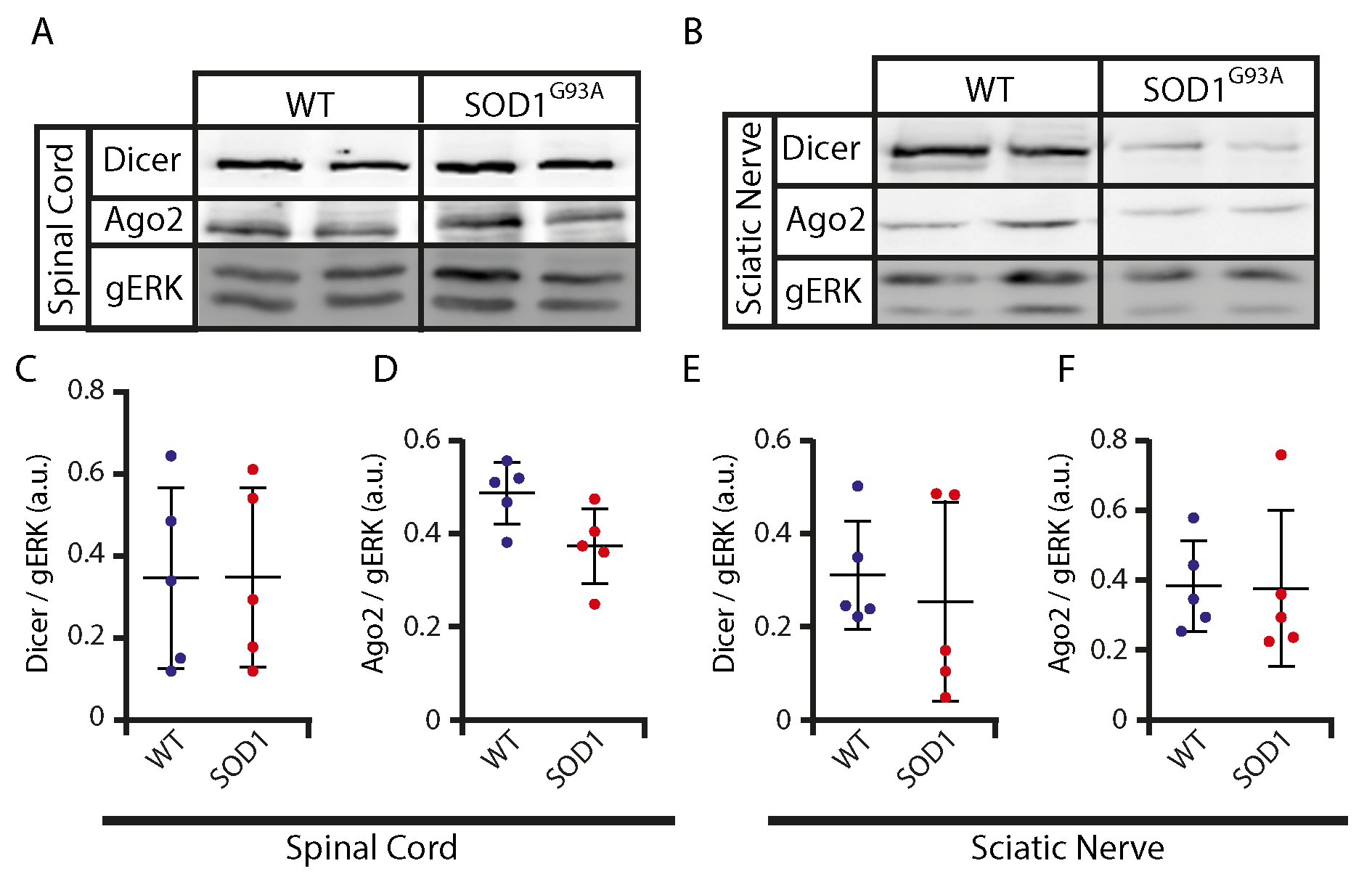

Supplement: Supplementary Figure 6 — SOD1 mutation causes no change in levels of RISC in mice tissues: (A,B) Western blot of extracts from five p120 mSOD1 and five littermate (LM) mouse spinal cords (A) and sciatic nerves (B) immunoblotted for Dicer and Ago2; gERK is used as a loading control. (C,D) Quantification of protein levels in spinal cord (n = 5) reveals no change in Dicer (C) and Ago2 (D) protein levels. (E,F) Quantification of protein levels in sciatic nerve (n = 5) reveals highly variable levels of Dicer (E) and no change in Ago2 (F) protein levels. [file Image_6.TIF]

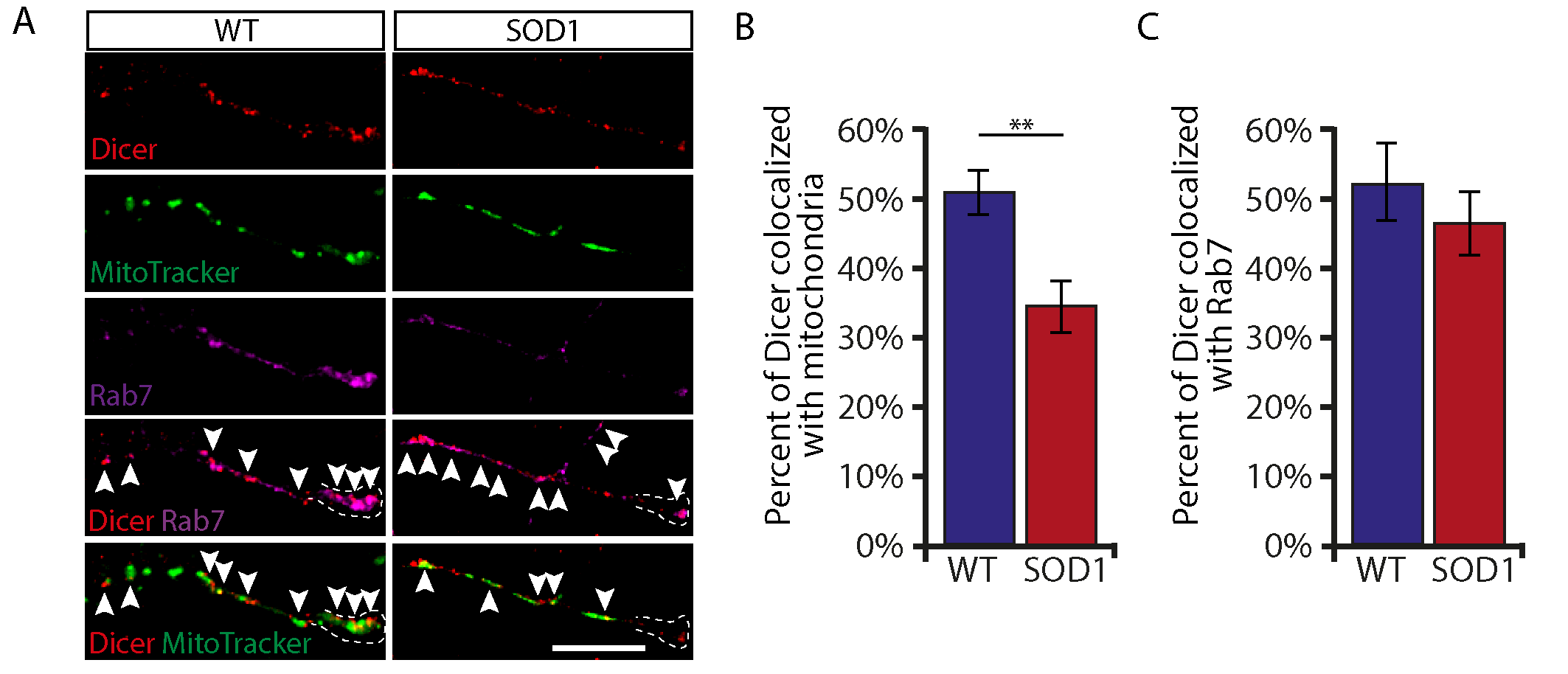

Supplement: Supplementary Figure 7 — SOD1 mutation causes reduced Dicer and mitochondria colocalization in MN: (A) Immunofluorescent images of primary motor neuron axons stained for Dicer (red), mitochondria (green), Rab7 (magenta). Overlay images show no difference in Rab7 and Dicer colocalization between WT and mSOD1 axons. Dicer colocalization with mitochondria is reduced in the growth cone (GC) in mSOD1 axons. The white dashed line indicates growth cone borders. (B,C) Colocalization analysis reveals a decreased colocalization of Dicer and mitochondria in mSOD1 neurons (B) but a similar colocalization of Dicer with Rab7 (C; **p < 0.01, n = 11 axons for WT, n = 11 axons for SOD1). Scale bar = 10 μm. [file Image_7.TIF]
